# Using Plant DNA Barcodes and Functional Traits to Assess Community Assembly of *Quercus* Forests at Different Scales in the Semiarid Loess Plateau of China

**DOI:** 10.1002/ece3.71103

**Published:** 2025-04-15

**Authors:** YongFu Chai, TingTing Tian, Luyao Wang, Junxin Wei, Yao Xu, Peiliang Liu, Chengcheng Xiang, Ming Yue

**Affiliations:** ^1^ Key Laboratory of Resource Biology and Biotechnology in Western China Xi'an Shaanxi China; ^2^ School of Life Sciences Northwest University Xi'an Shaanxi China

**Keywords:** community assembly, competitive exclusion, environment filtering, niche theories, temperate forest, vertical structure

## Abstract

Trait and evolutionary differences among coexisting species are increasingly used to comprehend the processes shaping communities. However, they do not consistently yield congruent insights due to methodological limitations and scale dependence. Utilizing two plastid DNA genes (rbcL and matK) and one nuclear DNA gene (internal transcribed spacer, ITS), we first constructed the phylogenies of 147 woody species from 98 line transects in the forest areas of the Loess Plateau and subsequently measured three functional traits. Five plots (2500 m^2^) were constructed within *Quercus* forests to analyze the functional and phylogenetic structures at three spatial scales (100, 400, 2500 m^2^) and two vertical structural layers (tree colonization and shrub layer). In contrast to the phylogenetic convergence observed at the genus level, using plant DNA barcodes, we found that the entire forest communities and the tree layer exhibited phylogenetic randomness across all three spatial scales; even the shrub layer showed phylogenetic overdispersion with increasing scale. Specific leaf area (SLA) exhibited functional convergence in both the shrub and tree layers. In contrast, seed mass (SM) and plant height (PH) displayed distinct functional structures. In the tree layer, these traits showed phylogenetic overdispersion, while in the shrub layer, they demonstrated functional convergence. This contrast highlights the different ecological roles and processes at play in the two layers. Specifically, the scale dependency of assembly patterns in the shrub layer was more pronounced than in the tree layer for both functional and phylogenetic structures. Our findings underscore the significance of employing DNA barcodes to assess the phylogenetic structure of communities with closely related coexisting species and emphasize niche‐based functional assembly and multi‐process phylogenetic assembly among vertical structural layers in the *Quercus* community. Decoupling functional and phylogenetic disparities between species could facilitate the understanding of complex species differences influencing community assembly.

## Graphical abstract

In contrast to phylogenetic convergence at the genus level, we found that the entire forest communities and tree layer exhibited phylogenetic randomness across all three scales, while the shrub layer demonstrated phylogenetic overdispersion with increasing scale. Specifically, the scale dependency of assembly patterns in the shrub layer was more pronounced than that in the tree layer for both functional and phylogenetic structures.

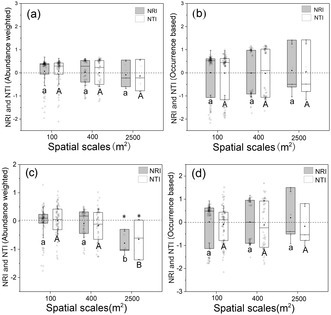

## 1 Introduction

Understanding the mechanisms of species co‐occurrence and biodiversity maintenance in natural forests is a central question in ecology (Ponisio et al. 2019). Neutral processes (random demographic dynamics) and niche‐based processes (environmental filtering and competitive exclusion) are considered the primary drivers of community assembly (Hillerislambers et al. 2012; Bañares‐de‐Dios et al. 2020). Although the contribution of these processes to community assembly has been demonstrated in various forests (Marteinsdóttir et al. 2018; Xiao et al. 2024; Zhang et al. 2015), few studies have explored whether these findings reflect the intrinsic nature of plant communities or result from methodological limitations. Addressing this question requires more comprehensive surveys employing standardized methods (Götzenberger et al. 2012).

The patterns of species coexistence in plant communities result from the interplay between species' evolutionary history and ecological processes (Huang and Zheng 2010; Webb 2000). Two approaches have been developed to account for the relative importance between neutral processes, niche‐based environmental filtering, and niche‐based competition exclusion in shaping community compositions: one based on evolutionary history (phylogenetic diversity) (Qian and Jin 2021) and another based on trait differences (functional diversity) (E‐Vojtkó et al. 2023). While these approaches are often assessed independently, a growing number of studies integrate both to infer community assembly mechanisms, as each approach involves distinct assumptions and limitations and may yield incongruent insights. A recent meta‐analysis revealed that phylogenetic and functional dispersion patterns were congruent in only about half of the cases (Cadotte et al. 2019), challenging the traditional hypothesis that community phylogeny can reliably predict the functional dispersion of woody plants (Jiang et al. 2018; Yang et al. 2014).

One reason for these incongruencies is that phylogenetic trees are often constructed using phylogenies resolved at the family level, and in some cases at the genus level, with genera and species added as polytomies to facilitate rapid detection of community structures (Qian and Jin 2021; E‐Vojtkó et al. 2023; Qin et al. 2017). The resulting tree topology, characterized by bifurcations and a lack of node support, may reduce the statistical power to detect non‐random patterns of community structure (Kress et al. 2009; Pei et al. 2011). DNA barcoding, besides its application in species identification and the discovery of cryptic species (Hebert et al. 2004), provides new opportunities and ideas to solve this problem and has a potential role in community phylogenies (Bennett et al. 2013; Chen et al. 2018; Erickson et al. 2014). Key DNA barcodes, such as plastid genes (rbcL and matK) and the nuclear gene internal transcribed spacer (ITS), are increasingly used to infer community assembly rules (Liu et al. 2019). For instance, niche‐related processes have been identified as the primary drivers of community composition in temperate forests (Chen et al. 2018; Erickson et al. 2014), whereas random processes predominantly shape communities in tropical forests (Swenson 2012). However, there are about 357,000 vascular plant species in the world, but only ∼20% of these species have been sequenced, according to gene sequence data with GenBank (Freiberg et al. 2020). Well‐resolved phylogenies that include all plant species of a study area are rare. So far, few studies have explored community phylogenetic structure by DNA barcodes in resource‐limited ecosystems such as the semiarid Loess Plateau, where climax forest communities often have more closely related species and similar species composition. Molecular identification is necessary to assess the difference of closely related species and infer community phylogenetic structure.

Another important consideration is that phylogenetic and functional information capture species' ecological differences unevenly. Phylogenies often better reflect multivariate conserved elements of ecological similarity, while single traits are more effective in capturing recent divergences, both of which influence ecological patterns (Cadotte et al. 2019). This underpins the phylogenetic niche conservatism hypothesis, which posits that closely related species tend to retain similar ecological characteristics within a community (Zhang et al. 2020). When combining phylogenetic and functional approaches to infer community processes, it is essential to assess the phylogenetic signals of functional traits under a Brownian motion model of evolution. In such cases, the combination of well‐resolved phylogenetic information and functional traits can enhance our ability to test community assembly theories (Yang et al. 2014).

The scale‐dependency of community assembly patterns reflects the influence of different ecological processes on community phylogenetic and functional structures (Cavender‐Bares et al. 2009). Previous studies in tropical and temperate forests have highlighted the importance of scale dependency in assessing community assembly processes, typically using either a phylogeny‐based approach (Ren et al. 2014) or a traits‐based approach separately (Yuan et al. 2016a). Generally, biotic interactions play a more significant role at smaller scales, whereas habitat filtering dominates at larger scales (Yang et al. 2014; Parmentier et al. 2014). However, few studies have combined well‐resolved phylogenetic information (e.g., DNA barcodes) and functional traits to investigate the scale dependency of community assembly processes within the same forest ecosystem (Yang et al. 2014), particularly in temperate forests. Temperate forests in harsh habitats often exhibit a simple composition of dominant species in the canopy layer but relatively high species diversity in the understory (Gilbert and Lechowicz 2004). This pattern suggests that different community assembly processes and scale‐dependent effects may shape species composition in the canopy and understory layers, whether assessed through community phylogenetic or functional structures.

The Loess Plateau in China, with an area of 62.4 × 104 km^2^, is considered one of the most seriously water‐eroded areas in the world (Wang et al. 2018; Yan et al. 2018). Ziwuling natural reserve (406.21 km^2^) is the major forest region on the Loess Plateau with extensive secondary deciduous broad‐leaved forests, and the climax forest community is dominated by *Quercus* species. The phylogenetic structure of the *Quercus* forest in this area has been studied at the genus level, lacking the well‐resolved phylogenies at the species level (Chai et al. 2016a). The objectives of this study are to (1) analyze phylogenetic and functional structures of *Quercus* forests by the combination of core DNA barcodes (rbcL, matK, and ITS) and plant traits; (2) disentangle the relative importance of niche‐based (environmental filtering and competitive exclusion) and neutral processes in driving community composition; and (3) elucidate the scale‐dependency of assembly pattern among different vertical structural layers. Answering these questions will help to provide a new perspective for future research on species coexistence and biodiversity conservation in water‐limited temperate deciduous broad‐leaved forests.

## 2 Materials and Methods

### 2.1 Study Area

The Loess Plateau (33°–41° N, 100°–114° E) in China has a continental monsoon climate, an average altitude between 1000 and 1500 m, and is severely affected by soil erosion. The annual average temperature is 3.6°C–14.3°C, and the annual precipitation is between 150 and 750 mm, while 60%–80% of the annual precipitation occurs from July–September (Zhang et al. 2016). The Ziwuling natural reserve of the Loess Plateau has the main natural secondary forest on the Loess Plateau (Zhao et al. 2016). The *Quercus* forest is the climax stage of the forest restoration succession and is important in soil and water conservation (Chai et al. 2016b).

### 2.2 Field Sampling and Functional Trait Data Collection

In order to construct the regional species pool of the forest community in the Ziwuling region, we determined the geographically predominant species based on the Floras (Liu 1998; Fu 2000), the Vegetation map of the People's Republic of China (Zhang 2007), and our previous investigation data (Wang et al. 2019). The line transect method of systematic sampling was used to collect specimens of the woody species in the distribution area of the forest. A total of 98 line transects (1.5 km per transect) were established, and 147 woody species were collected to represent the regional species pool of the forest community in the Ziwuling.

According to the distribution area of the *Quercus* forest, five plots with small disturbance and good development were located in the distribution area (Figure S1). The size of each forest plot was 2500 m^2^ (50m × 50 m), and the spatial distance between two plots was more than 2 km. Previous research has shown that 100 m^2^ is the smallest plot area for the survey of community species composition in temperate forests (Cavender‐Bares et al. 2006; Swenson et al. 2007); we thus divided the plot into twenty‐five 10 m × 10 m quadrats as the smallest scale. All trees (diameter at breast height > 1 cm) and shrubs were identified, and their maximum and average height, abundance, and coverage were documented. Finally, a total of 51 species were identified in the five plots. The size of the species pool may affect the result of community phylogenetic and functional structure when using a Null model. In most studies, the species pool is typically defined by all the species found in the sampled plots (51 species in our study). However, the number of species in the sampled plots may not adequately represent the regional species pool. To more accurately estimate assembly rules, we assessed the functional and phylogenetic structures respectively based on the plot‐specific species pool (composition of species that exist in the five plots, 51 species) and regional species pools (147 species).

Three functional traits, specific leaf area (SLA, a trade‐off between the leaf lifespan and lower construction cost), plant height (PH, a trade‐off between continuous access to light and early colonization), and seed mass (reproductive and dispersal abilities of species) were measured to represent the competitive strategy among coexisting species in forest ecosystems (Marteinsdóttir et al. 2018; Kunstler et al. 2016). The three traits were measured for all 147 species using standardized protocols (Cornelissen et al. 2003). For SM, the trait data mainly came from field samples; for species that did not have any seeds in the field, the data were obtained from floras (Liu 1998; Fu 2000) or the Kew Gardens database (www.rbgkew.org.uk/data/sid/).

### 2.3 Phylogenetic Reconstruction and Analysis

#### 2.3.1 Tissue Collection, Amplification, and Sequencing

Tissue samples were field collected and preserved via either flash freezing or silica gel desiccation. The genomic DNA was extracted from finely powdered sample material using the BioTeke Plant DNA extraction kit (Beijing). DNA barcodes were generated for the 147 species representing 21 orders, 41 families, and 85 genera. The abundance of the plant families among the 147 species is shown in Figure S2. For each species, sequences of the two plastid DNA genes (rbcL and matK) and one nuclear DNA gene (internal transcribed spacer, ITS) were obtained. The PCR protocols were as follows: for the rbcL region, 95°C for 4 min, 35 cycles of 94°C for 30 s, 54°C for 1 min, 72°C for 1 min, with a final extension of 72°C for 10 min; for the matK region, 95°C for 5 min, 38 cycles of 94°C for 30 s, 52°C for 1 min, 72°C for 1 min, with a final extension of 72°C for 7 min; and for the ITS region, 94°C for 5 min, 37 cycles of 94°C for 40 s, 60°C for 30 s, 72°C for 1 min, with a final extension of 72°C for 7 min. Experimental details of the primers are provided in Table S1, and we sent the amplified samples to the TSINGKE Company (China) for sequencing.

#### 2.3.2 Phylogenetic Reconstruction and Analyses

First, a species‐level phylogeny was generated based on DNA barcodes. Briefly, bidirectional trace files for each locus of the three markers were trimmed and assembled into contigs, and MUSCLE methods were used to obtain an alignment in Geneious V 8.0.2 (Bennett et al. 2013). Prior to inferring a maximum likelihood (ML) phylogeny via RAxML V 8.1.20 (Stamatakis et al. 2008) using the APG III phylogenetic tree as a constraint or guide tree to retain deep ancient nodes (Muscarella et al. 2014), the DNA supermatrix was generated from three loci through Sequence Matrix 1.7.8 (Rudolf et al. 2006). The APG classification is now updated as APG IV (2016), but the differences between APG III and APG IV are small. Finally, an ultra‐metric tree was calibrated using the penalized likelihood approach in the r8S software package (Sanderson 2003), placing constraints on the divergence times for the crown and stem groups at all 11 nodes (Lu et al. 2018) spread across the phylogenetic tree (Table S2). In addition, we generated a genus‐level phylogeny such that all the species within a genus have the same node ages (Qian and Jin 2021).

To test the scale dependence of the phylogenetic structure for forest communities, we divided the five 50 m × 50 m plots into sub‐quadrats with variable areas: 10 m × 10 m quadrats and 20 m × 20 m quadrats. The phylogenetic dispersion of the assemblages was quantified by the net relatedness index (NRI) and the net nearest taxa index (NTI). NRI and NTI are the standardized effect sizes (SES) for the mean pairwise phylogenetic distance (MPD) and the mean nearest taxon distance (MNTD) for all species in each quadrat. Taking account of the effects of species occurrence probability on community assembly, NRI and NTI were respectively calculated using the abundance‐weighted or occurrence‐based community data (Freilich and Connolly 2015). Null models were used in 999 randomizations to standardize the MPD and MNTD of each survey plot using the independent swap algorithm while maintaining per‐quadrat species richness and the frequency of species occurrence among the quadrats. NRI and NTI were calculated as follows (Swenson et al. 2007; Webb et al. 2002):

(1) $${\mathrm{NRI}}_{\text{sample}}=-1\times \frac{{\mathrm{MPD}}_{\text{sample}}-{\mathrm{MPD}}_{\text{randsample}}}{{\mathrm{SD}}_{1}\;\left({\mathrm{MPD}}_{\text{randsample}}\right)}$$

(2) $${\mathrm{NTI}}_{\text{sample}}=-1\times \frac{{\text{MNTD}}_{\text{sample}}-{\text{MNTD}}_{\text{randsample}}}{{\mathrm{SD}}_{2}\;\left({\text{MNTD}}_{\text{randsample}}\right)}$$

where MPD_sample_ and MNTD_sample_ represent the observed values, MPD_randsample_ and MNTD_randsample_ are the equivalents of the average values of the species obtained by randomization of the phylogenetic tree, and SD_1_ and SD_2_ are the standard deviations of the null distribution. A negative NRI or NTI indicates that a community is phylogenetically overdispersed (i.e., species are more distantly related than expected by chance), whereas a positive NRI or NTI value indicates phylogenetic clustering (i.e., species are more closely related than expected by chance), and the third condition is phylogenetically random distribution (species relations are consistent with those expected by chance). We use the ses.mpd and ses.mntd functions of the ‘Picante’ package (Kembel et al. 2010) in R v3.1.0 to calculate the NRI and NTI indices. Afterward, the barcode NRI or NTI values of all sub‐quadrats at each scale were averaged and compared to the expectation values of zero using a two‐tailed t‐test to determine the patterns of the community assembly at the scale level. The t‐test was performed in STATISTICA 10.0.

### 2.4 Analysis of Functional Trait Similarity and Phylogenetic Signals

Here, we used the mean functional trait distance (MFD) of pairwise species to calculate the functional structure of a quadrat in a community (Webb 2000). A standard effect size for each quadrat by comparing the observed MFD (FD_SES_) to 999 values generated by randomization to quantify the functional dispersions of the co‐occurring species was calculated as follows:

(3) $${\mathrm{FD}}_{\text{standard effect size}}=\frac{{\mathrm{MFD}}_{\text{sample}}-{\mathrm{MFD}}_{\text{randsample}}}{\mathrm{SD}\left({\mathrm{MFD}}_{\text{randsample}}\right)}$$

where SD stands for the standard deviation of the null distribution. A positive FD_SES_ value indicates trait divergence in a quadrat, whereas a negative FD_SES_ indicates that the traits are clustered in a quadrat. In total, all functional dispersion analyses were implemented in the ‘Picante’ package in R v3.1.0, and for each sub‐quadrat, the two‐tailed *t*‐test was used to assess the significant deviations of the FD_SES_ from the expected zero value.

To quantify the degree to which the phylogenetic tree predicts the functional similarity of the species, we calculated the phylogenetic signals of the three continuous traits by using Blomberg's *K* (Blomberg et al. 2003). Testing the significance of the *K* scores, the analysis was performed with 999 repetitions to generate a null distribution from which a *p* value could be calculated in the R package ‘phytools’ (Revell 2011).

## 3 Results

### 3.1 Sequencing, Species Identification, and Phylogenetic Reconstruction

The PCR and sequencing success rates were 100% for the rbcL region, while they were, respectively, 94.56% and 97.96% for the ITS and matK regions at the species level (Table S3). For the matK gene, three gymnosperm species failed to amplify; for ITS, the poor sequencing quality for a part of the taxa led to incomplete rates for sequence recovery (94.56%). For species with failed sequencing, we obtained corresponding ITS and matK sequence data from GenBank (Table S4). The barcode tree with optimal topology was the three‐locus supermatrix tree reconstructed using the Maximum Likelihood algorithm that applied the APG phylogenetic tree as a constrained tree.

To quantify the branch support of the Maximum‐Likelihood tree (ML tree), we defined four separate categories of parsimony ratchet values for each node of the tree: ≥ 85% = strong; 70%–85% = moderate; 50%–70% = weak; < 50% = poor or no support. The total fraction of nodes supported at each level was as follows: strong = 80.00%; moderate = 4.14%; weak = 8.97%; poor = 6.89% (Figure S3). The most fully resolved ML barcode tree (three‐locus supermatrix) supplied a 100% resolution at the species level for the quantification of accurate phylogenetic distances among the taxa in species pools (Figure S3).

### 3.2 Phylogenetic Structures of the Whole Community

**FIGURE 1:**
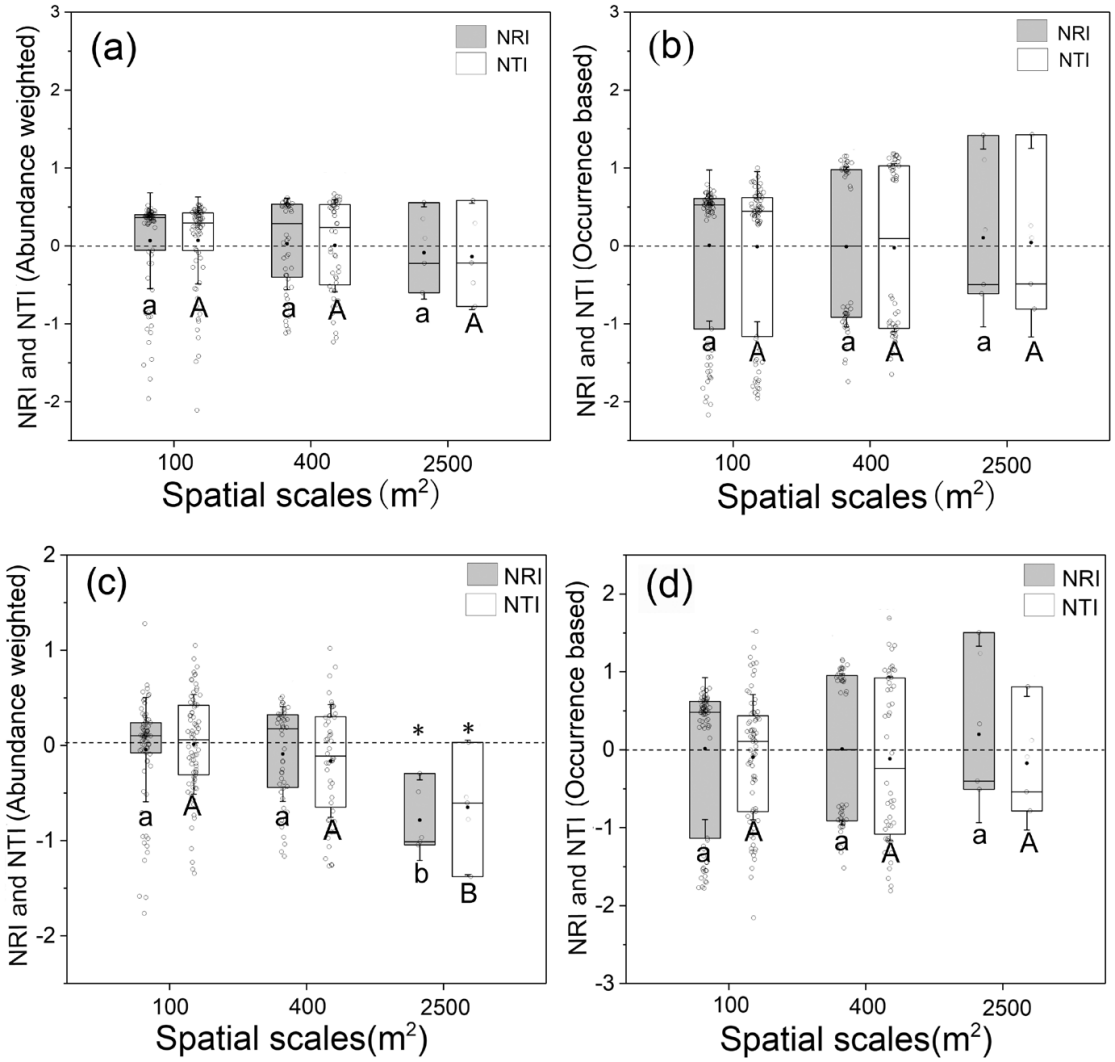
The net relatedness index (NRI) and the net nearest taxa index (NTI) of the whole community at different spatial scales using the region species pool (*n* = 147) (a, b) and the plot species pool (*n* = 51) (c, d). The asterisk indicates that the NRI or NTI is significantly different from null models (*p* < 0.05), while ns is not significantly different. NRI or NTI < 0 represents phylogenetic divergence, while NRI or NTI > 0 indicates phylogenetic convergence. Different letters represent significant differences in the phylogenetic structures among different spatial scales. The solid black line across the box represents the median value, and the black dot stands for the mean value. The blank circle on the boxes represents distribution of the data.

We applied the three‐locus barcode trees to perform the subsequent analysis of the community phylogenetic structures. Neutral community assemblage was the most common pattern at all spatial scales, either with the abundance‐weighted or no abundance‐weighted method, and no matter whether regional species pools (*n* = 147) or plot species pools (*n* = 51) were used (Figure 1). The only significant overdispersion pattern was the abundance‐weighted NRI based on the plot species pool at the largest spatial scales (Figure 1c). Moreover, we found a significant scale dependency of abundance‐weighted NRI and NTI based on the plot species pool (Figure 1c). Interestingly, we found that the results based on DNA barcode were entirely different from the results at the genus level, which revealed significant clustering of phylogenetic structure for the *Quercus* forest, either for the regional species pool or for the plot species pool (Tables S5 and S6).

### 3.3 Phylogenetic Structures of the Different Layers

**FIGURE 2:**
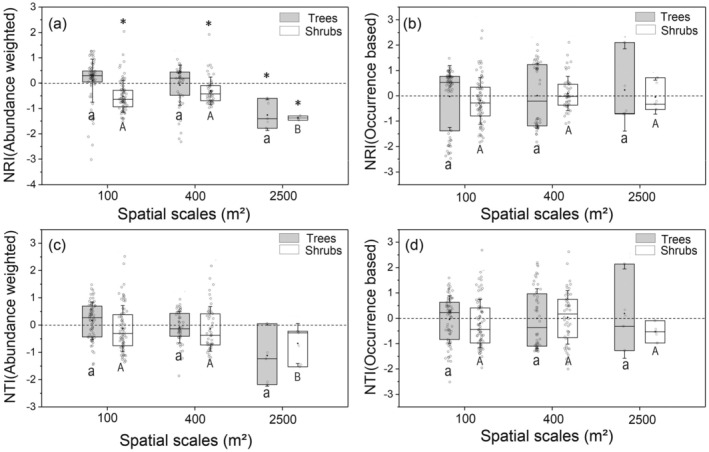
The net relatedness index (NRI) and the net nearest taxa index (NTI) of different vertical structural layers using the region species pool (*n* = 147) at three spatial scales. NRI or NTI of abundance weighted (a, c) and occurrence data‐based index (b, d) was estimated separately for the tree and shrubs layer. The asterisk indicates that the NRI is significantly different from null models (*p* < 0.05), while ns is not significantly different. NRI or NTI < 0 represents phylogenetic divergence, while NRI or NTI > 0 indicates phylogenetic convergence. Different letters represent significant differences in the phylogenetic structures among different spatial scales. The solid black line across the box represents the median value, and the black dot stands for the mean value. The blank circle on the boxes represents the distribution of the data.

**FIGURE 3:**
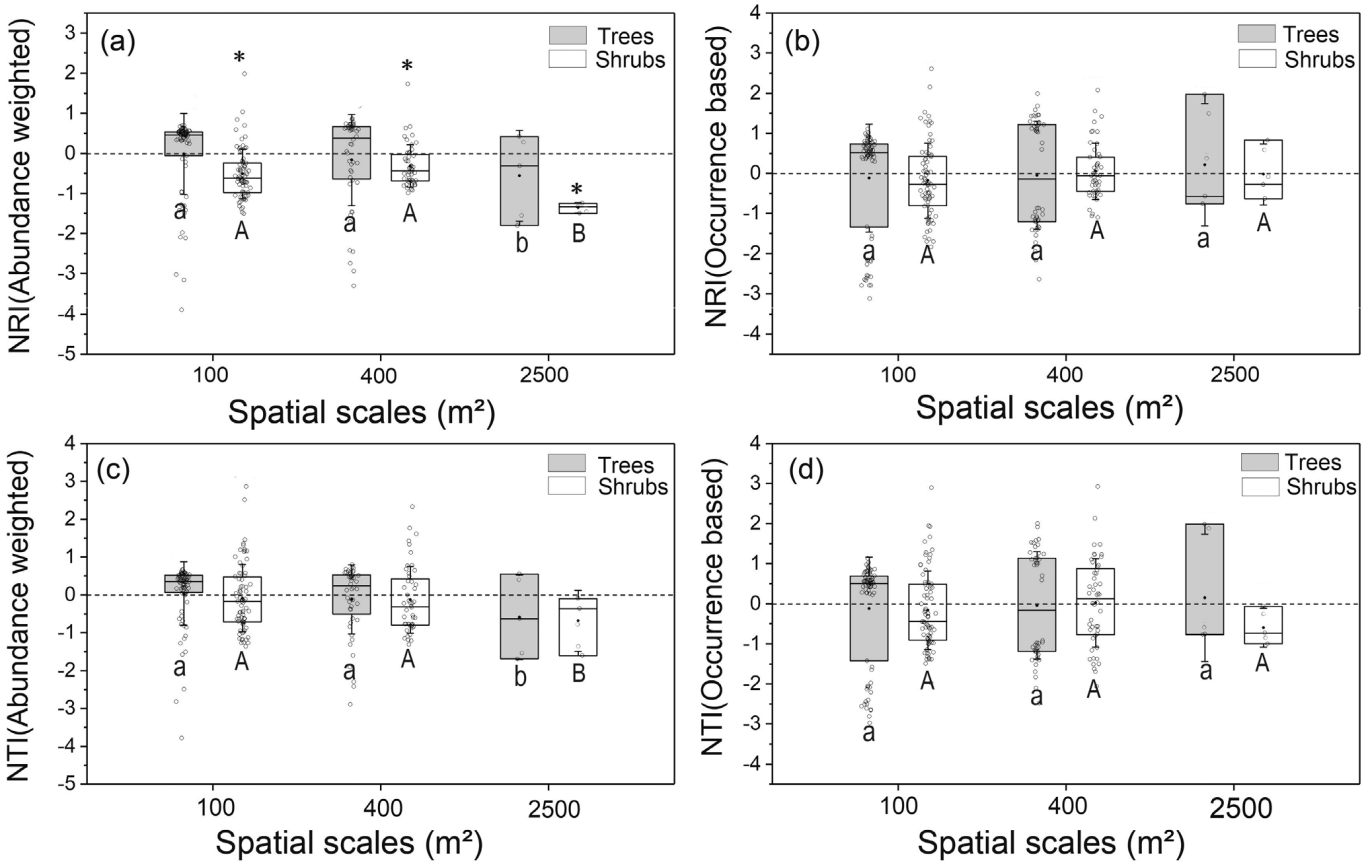
The net relatedness index (NRI) and the net nearest taxa index (NTI) of different vertical structural layers at three spatial scales using the plot species pool (*n* = 51). NRI or NTI of abundance weighted (a, c) and occurrence data‐based index (b, d) was estimated separately for the tree and shrubs layer. The asterisk indicates that the NRI is significantly different from null models (*p* < 0.05), while ns is not significantly different. NRI or NTI < 0 represents phylogenetic divergence, while NRI or NTI > 0 indicates phylogenetic convergence. Different letters represent significant differences in the phylogenetic structures among different spatial scales. The solid black line across the box represents the median value, and the black dot stands for the mean value. The blank circle on the boxes represents the distribution of the original data.

We then analyzed the phylogenetic structures of the tree and shrub layer of the *Quercus* forest separately and found that phylogenetic randomness was still the most common pattern (Figures 2 and 3). Conversely, the abundance‐weighted NRI of the shrub layer showed significant phylogenetic overdispersion at all spatial scales (Figures 2 and 3) based on either the regional species pool or the plot‐specific species pool. Moreover, the abundance‐weighted NRI of the shrub layer showed significant scale dependency; more phylogenetic overdispersion was found with increasing scale (Figures 2 and 3).

### 3.4 Functional Structures of Whole Community and Different Layers

**FIGURE 4:**
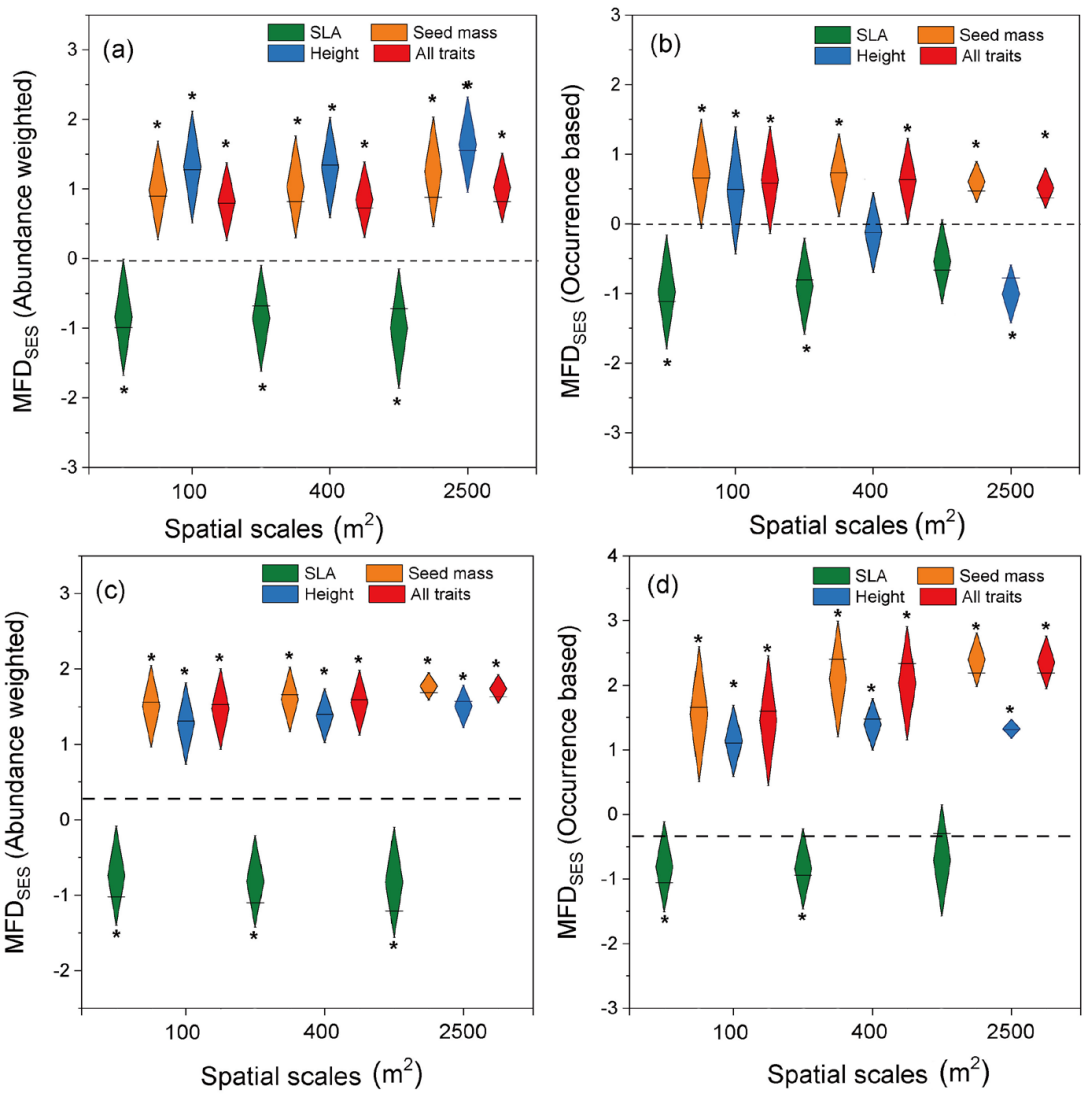
Standard effect sizes (SES) of functional trait distance (FD) for the whole community at different spatial scales using the region species pool (*n* = 147) (a, b) and the region species pool (*n* = 51) (b, d). Abundance weighted index (a, c) and occurrence data‐based index (b, d) were estimated separately. SLA: Specific leaf area. FDses > 0 represents divergence of community functional structure; FDses < 0 represents convergence in community functional structure. An asterisk indicates functional dispersion is significantly different from null values (*p* < 0.05), while ns is not significantly different.

**FIGURE 5:**
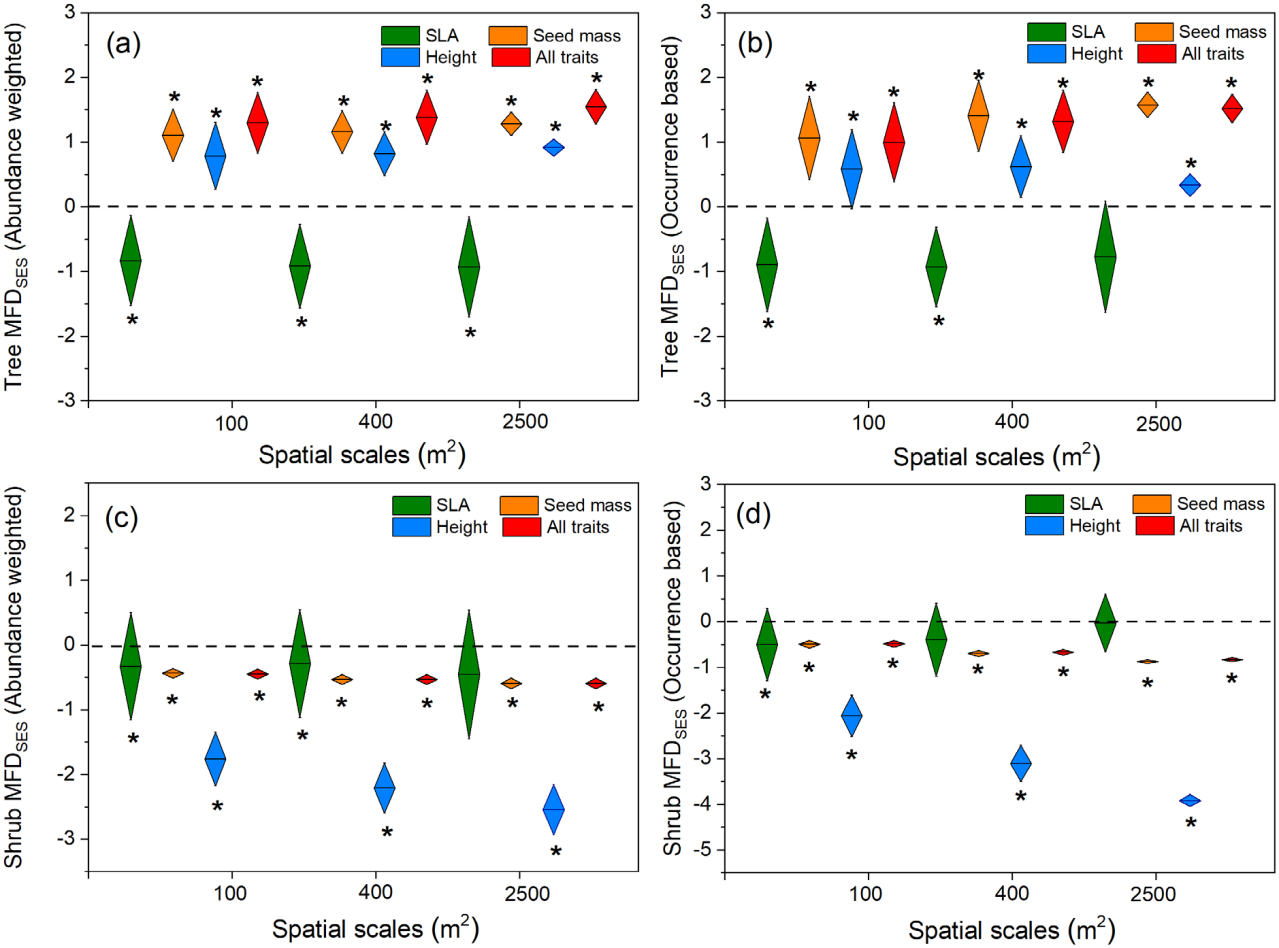
Standard effect sizes (SES) of functional trait distance (FD) for the tree layer and the shrub layer at different spatial scales using the region species pool (*n* = 147). Abundance weighted index (a, c) and occurrence data‐based index (b, d) were estimated separately. SLA: Specific leaf area. FDses > 0 stands for divergence in community functional dispersion; FDses < 0 stands for convergence in community functional dispersion. An asterisk indicates functional dispersion is significantly different from null values (*p* < 0.05), while ns is not significantly different.

**FIGURE 6:**
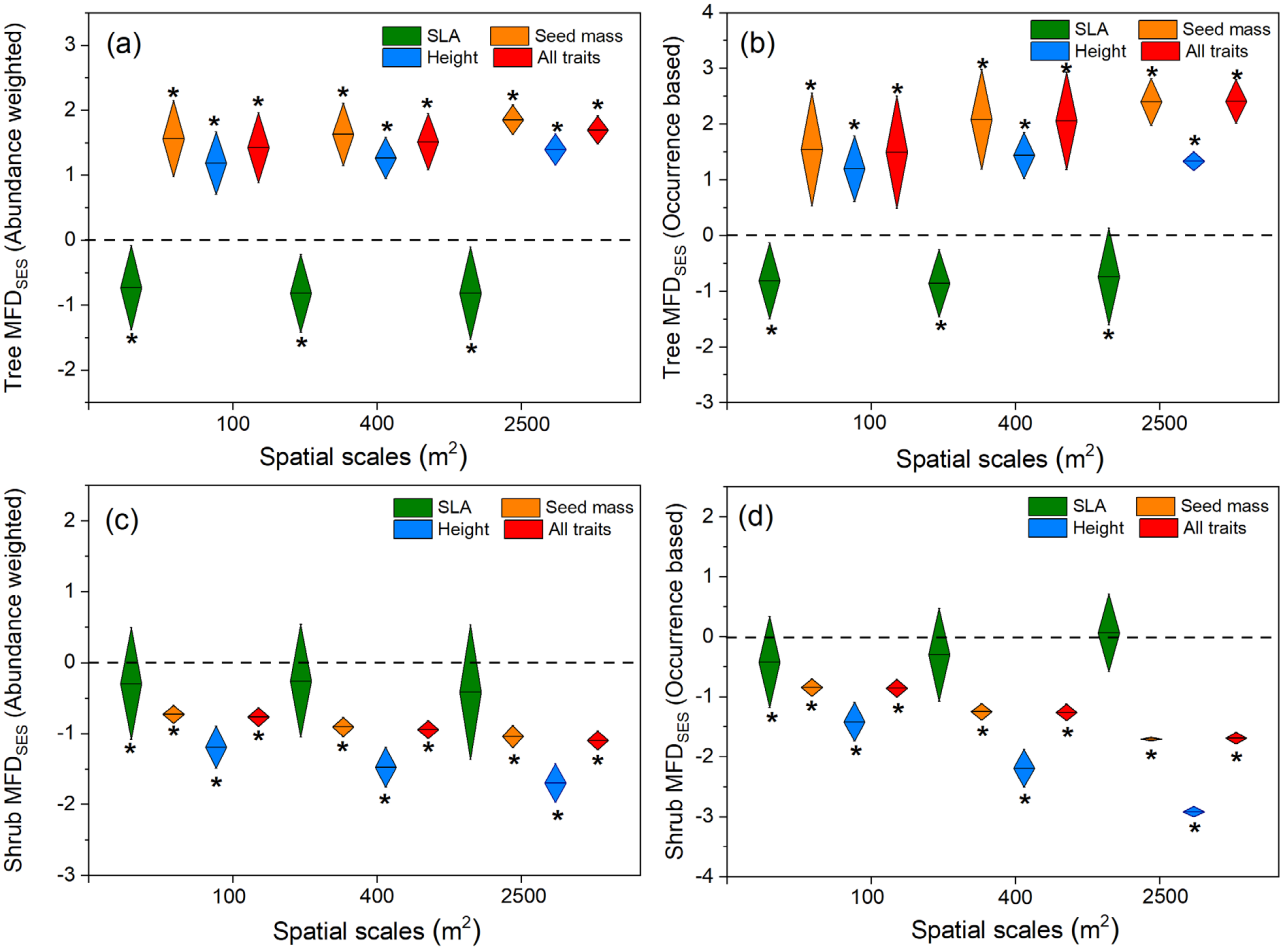
Standard effect sizes (SES) of functional trait distance (FD) for tree layer and shrub layer at different spatial scales using the plot species pool (*n* = 51). Abundance weighted index (a, c) and occurrence data‐based index (b, d) were estimated separately. SLA: Specific leaf area. FDses > 0 stands for divergence in community functional dispersion; FDses < 0 stands for convergence in community functional dispersion. The asterisk indicates functional dispersion is significantly different from null values (*p* < 0.05), while ns is not significantly different.

The whole community and tree layer revealed similar functional structures at three scales (Figure 4). Specifically, trait divergence was found for seed mass, height, and all traits at three spatial scales, and trait convergence was found for the SLA (Figure 4). In contrast, the shrub layer showed significant functional clustering for the three traits (SLA, SM, PH) and all traits (Figures 5 and 6). Furthermore, the functional structure of the single trait (SLA, SM, PH) and multiple traits for the shrub layer varied significantly with increasing spatial scales, either based on abundance weighted or occurrence data (Figures 5 and 6 and Tables S7 and S8). Seed mass and plant height became more significantly functionally clustered with increasing spatial scales; SLA showed the opposite pattern (Tables S7 and S8). However, for the tree layer, only the functional structure of seed mass showed a significant but opposite change with the shrub layer with the increasing spatial scales (Tables S7 and S8). These results indicate that the distribution of plant height, seed mass, and SLA at the shrub layer has a more significant and different scale dependence compared with that at the tree layer.

### 3.5 Phylogenetic Signals of Traits

We found significant phylogenetic signals (*p* < 0.05, Table 1) for SLA, SM, and plant height. The *K* values of phylogenetic signals for the three traits were less than 1 (mean = 0.088; range = 0.015 to 0.226; Table 1), indicating that the phylogenetic conservation of functional traits may be weak.

**TABLE 1:** The ranges of functional traits and the tests of the phylogenetic signal using Blomberg's *K* statistic for traits.

Traits	Ranges	*K*	*n*	*p*
SLA (cm^2^ g^−1^)	30.39–440.71	0.015	147	0.020
Seed mass (g)	0.05–11822.30	0.226	147	0.002
Height (m)	0.45–8.97	0.024	147	0.005

Abbreviations: *n*, the number of species with traits data represented in the phylogeny; SLA, specific leaf area.

## 4 Discussion

### 4.1 Species Identification and Community Phylogenies

Our results demonstrated that the application of three‐locus (rbcL + matK +ITS) barcodes generated a well‐resolved resolution at the species level of the phylogenies of 147 woody species from the Loess Plateau. The ML tree was constrained by combining a family‐level phylomatic tree and a three‐locus supermatrix that provided a 100% resolution of the terminal branches (Figure S3). Consequently, the cooperation of the internal transcribed spacer (ITS) region ameliorated the resolution of the species (Cheng et al. 2016; Li et al. 2011) and provided precise phylogenies for the subsequent community analysis. In addition, all the chloroplast matK genes of the angiosperms, except for three gymnosperm species, were successfully amplified, which indicated that the matK regions had wide applicability in the woody plants on the Loess Plateau, especially the amplifier pairs named KIM that had excellent universality and efficiency (Li et al. 2011). The reason for the failure of the matK region amplifications in these species was that the amplification primers selected in this study had poor adaptability in gymnosperms.

Although the total proportion of strong and moderate ratchet support throughout the ML tree reached 84.14%, there was a small percentage of nodes with weak (8.97%) and poor support (6.89%) (Figure S3). Nevertheless, the resolution at the species level for the barcode trees was up to 100%, which provided sufficiently accurate phylogenetic relationships for subsequent analysis of the community structures. In summary, with the continuous improvement in DNA barcoding and sequencing technologies, the ability of the three‐locus barcodes to reconstruct a well‐resolved phylogeny of the woody plants in the Loess Plateau adds extra value in improving DNA barcode libraries of forest ecosystems.

### 4.2 Different Phylogenetic Structure of Whole *Quercus* Forests Between Genus and Species Levels Reflects Shifts in Ecological Assembly Rules at Different Evolutionary Depths

Based on DNA barcodes, our results indicate that the phylogenetic structure of the whole *Quercus* forest in the Loess Plateau showed a stochastic pattern (Figure 1). This was different from the results at the family and genus levels that revealed significant clustering of phylogenetic structure for the *Quercus* forest (Chai et al. 2016a; Wang et al. 2019). Such differences suggest a discrepancy in the assembly rules at different evolutionary depths or taxonomic levels.

Some previous studies for temperate forests have argued that the deterministic niche‐based theory can explain the assembly mechanisms of the woody plant communities better (Pei et al. 2011; Chen et al. 2018; Wang et al. 2013). The stochastic pattern at the species level may indicate a stochastic ecological process, dominated by limited migration that affected the process of species aggregation. The clustering of phylogenetic structure at the genus level indicates the importance of environmental filtering in the Loess Plateau. An alternative explanation of the stochastic process was that the environmental modifications and limited similarity based on the niche‐based model and stochastic factors worked at the same time on the processes of community assembly (Lowe and Mcpeek 2014; Kraft et al. 2015). In other words, we may observe the zero‐sum state of the community, which was the neutralization of the environmental filtering positive force and the competitive exclusion negative force (Lowe and Mcpeek 2014). We consider the second interpretation seems the most likely. First, the *Quercus* forest is the climax community of succession in the Loess Plateau, and the limiting similarity (competition exclusion) inevitably increases because of species replacement during succession. Second, water availability is always the limiting factor for the ecological system of the Loess Plateau and semiarid habitats, especially for woody plant species. Thus, no matter how the research scales changed, water availability was the long‐standing environmental limiting factor. Therefore, we infer that the two deterministic processes may simultaneously shape the climax of the forest community successions in the Loess Plateau, merely increased competition at the species level partially offsetting the effects of environmental filtering.

The scale dependency of phylogenetic structure varied with forest types (Yang et al. 2014; Swenson et al. 2007). Although the conclusions of the scale dependency were inconsistent, most studies found that the community phylogenetic structures tended toward phylogenetically clustered increases within the spatial scales (Bennett et al. 2013; Cavender‐Bares et al. 2009; Swenson et al. 2007). Generally, 100 m^2^ was regarded as the critical value of scale for the transformation of the community phylogenetic structure (Swenson et al. 2007). Even so, we did not find a transformation in the phylogenetic structures of the whole Quercus forests in the semiarid Loess Plateau, at least at a spatial scale of up to 2500 m^2^. We observed only a tendency for phylogenetic structures to shift toward divergent distributions with increasing spatial scales when using the plot species pool (Figure 1). One possible explanation is that there may be multiple critical scale thresholds for the transition of phylogenetic structures in different forest communities with varying species pools. Another possible reason is that the significance of different ecological processes varies simultaneously with environmental conditions and spatial scale (Hillerislambers et al. 2012; Graham et al. 2018). For example, the role of environmental filtering in community assembly depends on the steepness of the environmental gradient (Willis et al. 2010). Therefore, both environmental severity and species pool size should be considered simultaneously to provide more comprehensive evidence for understanding the scale dependency of phylogenetic structures.

### 4.3 Different Phylogenetic Structure Between the Tree and Shrub Layer Reflects Shifts in Ecological Assembly Rules Among Vertical Structures

Previous community assembly studies based on DNA barcodes have primarily focused on the overall structure of communities without differentiating the phylogenetic structures of various vertical layers. Our results reveal distinct phylogenetic structures between vertical layers (tree and shrub layers) within the Quercus community on the Loess Plateau. Both NRI and NTI indices indicate a stochastic assembly pattern in the tree layer at all spatial scales (Figure 2, Figure 3), whereas abundance‐weighted NRI values demonstrate significant phylogenetic overdispersion in the shrub layer across spatial scales (Figure 2, Figure 3). Moreover, the shrub layer exhibits stronger scale dependence compared to the tree layer.

The differing survival strategies of shrub and tree species may drive variations in assembly mechanisms among vertical structures. Theoretically, the overdispersion pattern observed in the shrub layer is generally attributed to competitive exclusion (Purschke et al. 2013). The dense canopy of tree species creates a shaded understory environment, leading to intense competition for light and nutrient resources between understory and canopy species (e.g., asymmetric light competition) (Coomes et al. 2011). Additionally, understory shrub species are more susceptible to disturbances caused by environmental heterogeneity (Kristiansen et al. 2012), resulting in higher phylogenetic beta diversity compared to the tree layer in the *Quercus* forest (Wang et al. 2019). Altogether, these factors contribute to the overdispersion pattern observed in co‐occurring shrub layer species.

In contrast, the random phylogenetic structure in the tree layer may primarily indicate a balance between the positive force of environmental filtering and the negative force of competitive exclusion. Furthermore, limited seed dispersal of tall canopy species may significantly influence their distribution patterns, alongside other stochastic processes such as pathogen prevalence and feeding behaviors (Chen and Nan 2015). For instance, rodent feeding exerts a notable impact on *Quercus* seeds within the community, reducing seedling replacement and increasing surface seed disappearance rates.

### 4.4 Functional Traits Provide Different Information Compared to Phylogeny

We observed distinct functional structures compared to the phylogenetic structures in the *Quercus* community on the Loess Plateau. Significant functional convergence or divergence in SLA, SM, and plant height was observed either for the whole community or within the tree layer (Figures 4, 5, 6), whereas the phylogenetic structure appeared random. Even within the shrub layer, the functional structure exhibited complete convergence, contrasting with its divergent phylogenetic structure. These results highlight entirely different assembly mechanisms based on the phylogenetic and functional traits. Evidently, niche‐based deterministic processes exerted strong influences on the functional structures of the plant community. The three functional traits (SLA, SM, and PH) displayed significant but weak (*K* < 1, Table 1) phylogenetic signals, suggesting that the three functional traits are more labile and may not be phylogenetically conserved in our study area. Although previous studies have proposed phylogenetic diversity as a proxy for predicting community functional structure (Jiang et al. 2018; Baraloto et al. 2012), its effectiveness largely depends on the strength of phylogenetic signals (Lemoine et al. 2016). The long‐term selection pressures, such as the semiarid climate and water limitation on the Loess Plateau, may have weakened the relationship between plant traits and phylogeny, potentially explaining the inconsistencies between community assemblages based on phylogenetic and functional traits.

We compared the dissimilarities of functional structures corresponding to different traits. SLA revealed significant functional convergences, whereas SM and PH revealed divergences for both the whole community and tree layers. These findings stand in notable contrast to Marteinsdóttir et al. (2018) who found that neutral processes influenced the functional structure of SLA in temperate forests and de Bello et al. (2012) who found that there was a functional divergence of SLA in temperate forests. SLA's sensitivity to environmental changes and the environmental filtering under the harsh conditions of the Loess Plateau likely drive the convergence of its functional structures. The divergent functional structures of the SM and PH were consistent with previous results in temperate forests (de Bello et al. 2012) and tropical forests (Swenson and Enquist 2009). PH and SM, as ecological traits directly linked to resource competition, colonization, and growth strategies, are strongly influenced by competitive exclusion (Wang et al. 2019). These results suggest that community assembly mechanisms in temperate forests may differ depending on the traits, reflecting various survival strategies (Götzenberger et al. 2012). The trade‐offs among multiple traits are necessary for coexisting species to adapt to harsh habitats while enhancing competitiveness to acquire resources.

The scale dependence of community assembly based on functional traits has been explored in temperate and tropical forest ecosystems (Yang et al. 2014; Yuan et al. 2016b), with findings generally supporting the hypothesis that habitat filtering predominates at larger scales, while biotic determinism plays a greater role at smaller scales. However, these studies primarily focus on tree species. In the present study, understory shrub species exhibited entirely opposite functional structures compared to the tree layer across all three scales, except for SLA. Moreover, the shrub layer showed significant convergence in functional structures for all traits even at the smallest scale (100 m^2^). This underscores the influence of habitat filtering on the functional structure of the shrub layer, with its effects becoming more pronounced at larger scales, indicating stronger scale dependency for the shrub layer than the tree layer. Shrub species are more susceptible to environmental factors strongly associated with the acquisition and utilization of multiple resources (Kristiansen et al. 2012). Consequently, the differing response strategies of shrub and tree species may drive variations in assembly mechanisms across vertical structural layers and spatial scales.

## Author Contributions

**YongFu Chai:** conceptualization (equal), writing – original draft (lead). **TingTing Tian:** conceptualization (equal), data curation (lead), investigation (equal), methodology (equal), writing – original draft (supporting). **Luyao Wang:** data curation (supporting). **Junxin Wei:** data curation (supporting). **Yao Xu:** investigation (equal), methodology (equal). **Peiliang Liu:** investigation (equal), methodology (equal). **Chengcheng Xiang:** writing – review and editing (supporting). **Ming Yue:** conceptualization (lead), funding acquisition (lead), writing – review and editing (lead).

## Conflicts of Interest

The authors declare no conflicts of interest.

## Supporting information


Appendix S1.

## Data Availability

The major datasets supporting the conclusions of this article will be archived in GenBank. DNA sequences: GenBank accessions MN721982—MN722410.

## References

[ece371103-bib-0001] Bañares‐de‐Dios, G. , M. J. Macía , Í. G. la Cerda , et al. 2020. “Linking Patterns and Processes of Tree Community Assembly Across Spatial Scales in Tropical Montane Forests.” Ecology 101: e03058. 10.1002/ecy.3058. 32304221

[ece371103-bib-0002] Baraloto, C. , O. J. Hardy , C. Paine , K. G. Dexter , and J. Chave . 2012. “Using Functional Traits and Phylogenetic Trees to Examine the Assembly of Tropical Tree Communities.” Journal of Ecology 100: 690–701. 10.1111/j.1365-2745.2012.01966.x.

[ece371103-bib-0003] Bennett, J. A. , E. G. Lamb , J. C. Hall , W. M. Cardinal‐McTeague , and J. F. Cahill . 2013. “Increased Competition Does Not Lead to Increased Phylogenetic Overdispersion in a Native Grassland.” Ecology Letters 16: 1168–1176. 10.1111/ele.12153. 23841858

[ece371103-bib-0004] Blomberg, S. P. , T. Garland , and A. R. Ives . 2003. “Testing for Phylogenetic Signal in Comparative Data: Behavioral Traits Are More Labile.” Evolution 57: 717–745. 10.1111/j.0014-3820.2003.tb00285.x. 12778543

[ece371103-bib-0005] Cadotte, M. W. , M. Carboni , X. Si , and S. Tatsumi . 2019. “Do Traits and Phylogeny Support Congruent Community Diversity Patterns and Assembly Inferences?” Journal of Ecology 107: 2065–2077. 10.1111/1365-2745.13247.

[ece371103-bib-0006] Cavender‐Bares, J. , A. Keen , and B. Miles . 2006. “Phylogenetic Structure of Floridian Plant Communities Depends on Taxonomic and Spatial Scale.” Ecology 87: S109–S122. 10.1890/0012-9658(2006)87[109:PSOFPC]2.0.CO;2. 16922307

[ece371103-bib-0007] Cavender‐Bares, J. , K. H. Kozak , P. Fine , and S. W. Kembel . 2009. “The Merging of Community Ecology and Phylogenetic Biology.” Ecology Letters 12: 693–715. 10.1111/j.1461-0248.2009.01314.x. 19473217

[ece371103-bib-0008] Chai, Y. , M. Yue , X. Liu , et al. 2016a. “Patterns of Taxonomic, Phylogenetic Diversity During a Long‐Term Succession of Forest on the Loess Plateau, China: Insights Into Assembly Process.” Scientific Reports 6: 27087. 10.1038/srep27087. 27272407 PMC4897607

[ece371103-bib-0009] Chai, Y. , M. Yue , M. Wang , et al. 2016b. “Plant Functional Traits Suggest a Change in Novel Ecological Strategies for Dominant Species in the Stages of Forest Succession.” Oecologia 180: 771–783. 10.1007/s00442-015-3483-3. 26563469

[ece371103-bib-0010] Chen, L. , L. S. Comita , S. J. Wright , et al. 2018. “Forest Tree Neighborhoods Are Structured More by Negative Conspecific Density Dependence Than by Interactions Among Closely Related Species.” Ecography 10: 1114–1123. 10.1111/ecog.03389.

[ece371103-bib-0011] Chen, T. , and Z. Nan . 2015. “Effects of Phytopathogens on Plant Community Dynamics: A Review.” Acta Ecologica Sinica 35: 177–183. 10.1016/j.chnaes.2015.09.003.

[ece371103-bib-0012] Cheng, T. , C. Xu , L. Lei , C. Li , Y. Zhang , and S. Zhou . 2016. “Barcoding the Kingdom Plantae: New PCR Primers for ITS Regions of Plants With Improved Universality and Specificity.” Molecular Ecology Resources 16: 138–149. 10.1111/1755-0998.12438. 26084789

[ece371103-bib-0013] Coomes, D. A. , E. R. Lines , and R. B. Allen . 2011. “Moving on From Metabolic Scaling Theory: Hierarchical Models of Tree Growth and Asymmetric Competition for Light.” Journal of Ecology 99: 748–756. 10.1111/j.1365-2745.2011.01811.x.

[ece371103-bib-0014] Cornelissen, J. , S. Lavorel , E. Garnier , et al. 2003. “A Handbook of Protocols for Standardised and Easy Measurement of Plant Functional Traits Worldwide.” Australian Journal of Botany 51: 335–380. 10.1071/BT02124.

[ece371103-bib-0015] de Bello, F. , J. N. Price , T. Münkemüller , J. Liira , M. Zobel , and W. Thuiller . 2012. “Functional Species Pool Framework to Test for Biotic Effects on Community Assembly.” Ecology 93: 2263–2273. 10.1890/11-1394.1. 23185887

[ece371103-bib-0016] Erickson, D. L. , F. A. Jones , N. G. Swenson , et al. 2014. “Comparative Evolutionary Diversity and Phylogenetic Structure Across Multiple Forest Dynamics Plots: A Mega‐Phylogeny Approach.” Frontiers in Genetics 5: 358. 10.3389/fgene.2014.00358. 25414723 PMC4220724

[ece371103-bib-0017] E‐Vojtkó, A. , F. de Bello , Z. Lososová , and L. Götzenberger . 2023. “Phylogenetic Diversity Is a Weak Proxy for Functional Diversity but They Are Complementary in Explaining Community Assembly Patterns in Temperate Vegetation.” Journal of Ecology 111: 2218–2230. 10.1111/1365-2745.14171.

[ece371103-bib-0018] Freiberg, M. , M. Winter , A. Gentile , et al. 2020. “LCVP, the Leipzig Catalogue of Vascular Plants, a New Taxonomic Reference List for all Known Vascular Plants.” Scientific Data 7: 416. 10.1038/s41597-020-00702-z. 33243996 PMC7693275

[ece371103-bib-0019] Freilich, M. A. , and S. R. Connolly . 2015. “Phylogenetic Community Structure When Competition and Environmental Filtering Determine Abundances.” Global Ecology and Biogeography 24: 1390–1400. 10.1111/geb.12367.

[ece371103-bib-0020] Fu, J. Q. 2000. Flora Loess‐Plateaus Sinicae. Science Press.

[ece371103-bib-0021] Gilbert, B. , and M. J. Lechowicz . 2004. “Neutrality, Niches, and Dispersal in a Temperate Forest Understory.” Proceedings of the National Academy of Sciences of the United States of America 101: 7651–7656. 10.1073/pnas.0400814101. 15128948 PMC419661

[ece371103-bib-0022] Götzenberger, L. , F. D. Bello , K. A. Bråthen , et al. 2012. “Ecological Assembly Rules in Plant Communities—Approaches, Patterns and Prospects.” Biological Reviews 87: 111–127. 10.1111/j.1469-185X.2011.00187.x. 21692965

[ece371103-bib-0023] Graham, C. H. , D. Storch , and A. Machac . 2018. “Phylogenetic Scale in Ecology and Evolution.” Global Ecology and Biogeography 27: 175–187. 10.1111/geb.12686.

[ece371103-bib-0024] Hebert, P. , E. H. Penton , J. M. Burns , D. H. Janzen , and W. Hallwachs . 2004. “Ten Species in One: DNA Barcoding Reveals Cryptic Species in the Neotropical Skipper Butterfly *Astraptes fulgerator* .” Proceedings of the National Academy of Sciences of the United States of America 76: 179. 10.1073/pnas.0406166101. PMC522015 15465915

[ece371103-bib-0025] Hillerislambers, J. , P. B. Adler , W. S. Harpole , J. M. Levine , and M. M. Mayfield . 2012. “Rethinking Community Assembly Through the Lens of Coexistence Theory.” Annual Review of Ecology, Evolution, and Systematics 43: 227–248. 10.1146/annurev-ecolsys-110411-160411.

[ece371103-bib-0026] Huang, J. X. , and F. Y. Zheng . 2010. “Influence of Environmental Factors on Phylogenetic Structure at Multiple Spatial Scales in an Evergreen Broad‐Leaved Forest of China.” Chinese Journal of Plant Ecology 34: 309–315. 10.3724/SP.J.1142.2010.40521.

[ece371103-bib-0027] Jiang, F. , Y. Xun , H. Cai , and G. Jin . 2018. “What Factors Potentially Influence the Ability of Phylogenetic Distance to Predict Trait Dispersion in a Temperate Forest?” Ecology and Evolution 8: 1107–1116. 10.1002/ece3.3691. 29375783 PMC5773330

[ece371103-bib-0028] Kembel, S. W. , P. D. Cowan , M. R. Helmus , et al. 2010. “Picante: R Tools for Integrating Phylogenies and Ecology.” Bioinformatics 26, no. 11: 1463–1464. 20395285 10.1093/bioinformatics/btq166

[ece371103-bib-0029] Kraft, N. J. B. , P. B. Adler , O. Godoy , E. C. James , S. Fuller , and J. M. Levine . 2015. “Community Assembly, Coexistence and the Environmental Filtering Metaphor.” Functional Ecology 29: 592–599. 10.1111/1365-2435.12345.

[ece371103-bib-0030] Kress, W. J. , D. L. Erickson , F. A. Jones , et al. 2009. “Plant DNA Barcodes and a Community Phylogeny of a Tropical Forest Dynamics Plot in Panama.” Proceedings of the National Academy of Sciences of the United States of America 106: 18621–18626. 10.1073/pnas.0909820106. 19841276 PMC2763884

[ece371103-bib-0031] Kristiansen, T. , J. C. Svenning , W. L. Eiserhardt , et al. 2012. “Environment Versus Dispersal in the Assembly of Western Amazonian Palm Communities.” Journal of Biogeography 39: 1318–1332. 10.1111/j.1365-2699.2012.02689.x.

[ece371103-bib-0032] Kunstler, G. , D. Falster , D. A. Coomes , et al. 2016. “Plant Functional Traits Have Globally Consistent Effects on Competition.” Nature 529: 204–207. 10.1038/nature16476. 26700807

[ece371103-bib-0033] Lemoine, N. P. , J. Shue , B. Verrico , D. Erickson , W. J. Kress , and J. D. Parker . 2016. “Phylogenetic Relatedness and Leaf Functional Traits, Not Introduced Status, Influence Community Assembly.” Ecology 96: 2605–2612. 10.1890/14-1883.1. 26649382

[ece371103-bib-0034] Li, D. Z. , L. M. Gao , H. T. Li , et al. 2011. “Comparative Analysis of a Large Dataset Indicates That Internal Transcribed Spacer (ITS) Should be Incorporated Into the Core Barcode for Seed Plants.” Proceedings of the National Academy of Sciences of the United States of America 108: 19641–19646. 10.1073/pnas.1104551108. 22100737 PMC3241788

[ece371103-bib-0035] Liu, J. , J. Liu , Y.‐X. Shan , X.‐J. Ge , and K. S. Burgess . 2019. “The Use of DNA Barcodes to Estimate Phylogenetic Diversity in Forest Communities of Southern China.” Ecology and Evolution 9: 5372–5379. 10.1002/ece3.5128. 31110686 PMC6509380

[ece371103-bib-0036] Liu, L. P. 1998. Flora Arborum ET Fruticum Ziwu Montium. Lanzhou University Press.

[ece371103-bib-0037] Lowe, W. H. , and M. A. Mcpeek . 2014. “Is Dispersal Neutral?” Trends in Ecology & Evolution 29: 444–450. 10.1016/j.tree.2014.05.009. 24962790

[ece371103-bib-0038] Lu, L. M. , L. F. Mao , T. Yang , J. F. Ye , B. Liu , and H. L. Li . 2018. “Evolutionary History of the Angiosperm Flora of China.” Nature 554: 234–238. 10.1038/nature25485. 29420476

[ece371103-bib-0039] Marteinsdóttir, B. , K. Svavarsdóttir , and T. E. Thórhallsdóttir . 2018. “Multiple Mechanisms of Early Plant Community Assembly With Stochasticity Driving the Process.” Ecology 99: 91–102. 10.1002/ecy.2079. 29121406

[ece371103-bib-0040] Muscarella, R. , M. Uriarte , D. L. Erickson , N. G. Swenson , J. K. Zimmerman , and W. J. Kress . 2014. “A Well‐Resolved Phylogeny of the Trees of Puerto Rico Based on DNA Barcode Sequence Data.” PLoS One 9: 112843. 10.7916/D80K27DX. PMC4227909 25386879

[ece371103-bib-0041] Parmentier, I. , M. Réjou‐Méchain , J. Chave , et al. 2014. “Prevalence of Phylogenetic Clustering at Multiple Scales in an African Rainforest Tree Community.” Journal of Ecology 102: 1008–1016. 10.1111/1365-2745.12254.

[ece371103-bib-0042] Pei, N. , J. Y. Lian , D. L. Erickson , et al. 2011. “Exploring Tree‐Habitat Associations in a Chinese Subtropical Forest Plot Using a Molecular Phylogeny Generated From DNA Barcode Loci.” PLoS One 6: e21273. 10.1371/journal.pone.0021273. 21701680 PMC3119057

[ece371103-bib-0043] Ponisio, L. C. , F. S. Valdovinos , K. T. Allhoff , et al. 2019. “A Network Perspective for Community Assembly.” Frontiers in Ecology and Evolution 7: 103. 10.3389/fevo.2019.00103.

[ece371103-bib-0044] Purschke, O. , B. C. Schmid , M. T. Sykes , et al. 2013. “Contrasting Changes in Taxonomic, Phylogenetic and Functional Diversity During a Long‐Term Succession: Insights Into Assembly Processes.” Journal of Ecology 101: 857–866. 10.1111/1365-2745.12098.

[ece371103-bib-0045] Qian, H. , and Y. Jin . 2021. “Are Phylogenies Resolved at the Genus Level Appropriate for Studies on Phylogenetic Structure of Species Assemblages?” Plant Diversity 43: 255–263. 10.1016/j.pld.2020.11.005. 34485767 PMC8390917

[ece371103-bib-0046] Qin, H. , G. Dong , Y. Zhang , F. Zhang , and M. Wang . 2017. “Patterns of Species and Phylogenetic Diversity of *Pinus tabuliformis* Forests in the Eastern Loess Plateau, China.” Forest Ecology and Management 394: 42–51. 10.1016/j.foreco.2017.03.030.

[ece371103-bib-0047] Ren, S. , T. Wang , Y. Zhu , et al. 2014. “Phylogenetic Structure of Individuals With Different DBH Sizes in a Deciduous Broad‐Leaved Forest Community in the Temperate‐Subtropical Ecological Transition Zone, China.” Biodiversity Science 22: 574. 10.3724/SP.J.1003.2014.14116.

[ece371103-bib-0048] Revell, L. J. 2011. “Phytools: An R Package for Phylogenetic Comparative Biology (And Other Things).” Methods in Ecology and Evolution 3: 217–223. 10.1111/j.2041-210X.2011.00169.x.

[ece371103-bib-0049] Rudolf, M. , S. Kwong , V. Gaurav , and P. Ng . 2006. “DNA Barcoding and Taxonomy in Diptera: A Tale of High Intraspecific Variability and Low Identification Success.” Systematic Biology 55: 715–728. 10.1080/10635150600969864. 17060194

[ece371103-bib-0050] Sanderson, M. J. 2003. “R8s: Inferring Absolute Rates of Molecular Evolution and Divergence Times in the Absence of a Molecular Clock.” Bioinformatics 19: 301–302. 10.1093/bioinformatics/19.2.301. 12538260

[ece371103-bib-0051] Stamatakis, A. , P. Hoover , and J. Rougemont . 2008. “A Rapid Bootstrap Algorithm for the RAxML Web Servers.” Systematic Biology 57, no. 5: 758–771. 10.1080/10635150802429642. 18853362

[ece371103-bib-0052] Swenson, N. G. 2012. “Phylogenetic Analyses of Ecological Communities Using DNA Barcode Data.” Methods in Molecular Biology 858: 409. 10.1007/978-1-61779-591-6_20. 22684968

[ece371103-bib-0053] Swenson, N. G. , and B. J. Enquist . 2009. “Opposing Assembly Mechanisms in a Neotropical Dry Forest: Implications for Phylogenetic and Functional Community Ecology.” Ecology 90: 2161. 10.1890/08-1025.1. 19739378

[ece371103-bib-0054] Swenson, N. G. , B. J. Enquist , J. Thompson , and J. K. Zimmerman . 2007. “The Influence of Spatial and Size Scale on Phylogenetic Relatedness in Tropical Forest Communities.” Ecology 88: 1770–1780. 10.1890/06-1499.1. 17645023

[ece371103-bib-0055] Wang, M. , J. Xu , Y. Chai , Y. Guo , X. Liu , and M. Yue . 2019. “Differentiation of Environmental Conditions Promotes Variation of Two Quercus Wutaishanica Community Assembly Patterns.” Forests 11, no. 1: 43. 10.3390/f11010043.

[ece371103-bib-0056] Wang, X. , N. G. Swenson , T. Wiegand , et al. 2013. “Phylogenetic and Functional Diversity Area Relationships in Two Temperate Forests.” Ecography 36: 883–893. 10.1111/j.1600-0587.2012.00011.x.

[ece371103-bib-0057] Wang, Y. , M. Brandt , M. Zhao , et al. 2018. “Major Forest Increase on the Loess Plateau, China (2001–2016).” Land Degradation & Development 29: 4080–4091. 10.1002/ldr.3174.

[ece371103-bib-0058] Webb, C. O. 2000. “Exploring the Phylogenetic Structure of Ecological Communities: An Example for Rain Forest Trees.” American Naturalist 156: 145–155. 10.1086/303378. 10856198

[ece371103-bib-0059] Webb, C. O. , D. D. Ackerly , M. A. McPeek , M. J. Donoghue , and J. Michael . 2002. “Phylogenies and Community Ecology.” Annual Review of Ecology and Systematics 33, no. 1: 475–505.

[ece371103-bib-0060] Willis, C. G. , M. Halina , C. Lehman , et al. 2010. “Phylogenetic Community Structure in Minnesota Oak Savanna Is Influenced by Spatial Extent and Environmental Variation.” Ecography 33: 565–577. 10.1111/j.1600-0587.2009.05975.x.

[ece371103-bib-0061] Xiao, C. , G. Feng , and W. Long . 2024. “Pattern and Driver of the Compositional Variations in a Tropical Cloud Forest: Comparing Vascular Epiphytes With Terrestrial Woody Plants.” Oikos 2024, no. 4: e10158. 10.1111/oik.10158.

[ece371103-bib-0062] Yan, R. , X. Zhang , S. Yan , J. Zhang , and H. Chen . 2018. “Spatial Patterns of Hydrological Responses to Land Use/Cover Change in a Catchment on the Loess Plateau, China.” Ecological Indicators 92: 151–160. 10.1016/j.ecolind.2017.04.013.

[ece371103-bib-0063] Yang, J. , G. Zhang , X. Ci , et al. 2014. “Functional and Phylogenetic Assembly in a Chinese Tropical Tree Community Across Size Classes, Spatial Scales and Habitats.” Functional Ecology 28: 520–529. 10.1111/1365-2435.12176.

[ece371103-bib-0064] Yuan, Z. , A. Gazol , F. Lin , et al. 2016b. “Scale‐Dependent Effect of Biotic Interactions and Environmental Conditions in Community Assembly: Insight From a Large Temperate Forest Plot.” Plant Ecology 217: 1003–1014. 10.1007/s11258-016-0626-5.

[ece371103-bib-0065] Yuan, Z. , S. Wang , A. Gazol , et al. 2016a. “Multiple Metrics of Diversity Have Different Effects on Temperate Forest Functioning Over Succession.” Oecologia 182: 1175–1185. 10.1007/s00442-016-3737-8. 27677471

[ece371103-bib-0066] Zhang, H. , W. Qi , R. John , W. Wang , F. Song , and S. Zhou . 2015. “Using Functional Trait Diversity to Evaluate the Contribution of Multiple Ecological Processes to Community Assembly During Succession.” Ecography 38: 1176–1186. 10.1111/ecog.01123.

[ece371103-bib-0067] Zhang, H. Y. , N. F. Fang , and Z. H. Shi . 2016. “Spatio‐Temporal Patterns for the NDVI and Its Responses to Climatic Factors in the Loess Plateau, China.” Acta Ecologica Sinica 36: 3960–3968. 10.5846/stxb201506281310.

[ece371103-bib-0068] Zhang, J. , N. G. Swenson , J. Liu , M. Liu , X. Qiao , and M. Jiang . 2020. “A Phylogenetic and Trait‐Based Analysis of Community Assembly in a Subtropical Forest in Central China.” Ecology and Evolution 10: 8091–8104. 10.1002/ece3.6465. 32788963 PMC7417225

[ece371103-bib-0069] Zhang, X. S. 2007. Vegetation Map of the People's Republic of China (1:1 000 000). 1st ed. Geology Press.

[ece371103-bib-0070] Zhao, M. , X. Feng , Y. LÜ , W. Zuo , and M. Kang . 2016. “The Effect of Environmental Factors on the Distribution of Litter Mass and Litter Depth in Forests of Loess Plateau Region.” Acta Ecologica Sinica 36: 7364–7373. 10.5846/stxb201601050026.

