# Supplementary material for: Using Plant DNA Barcodes and Functional Traits to Assess Community Assembly of Quercus Forests at Different Scales in the Semiarid Loess Plateau of China
Source: Ecol Evol. 2025 Apr 15;15(4):e71103. doi: 10.1002/ece3.71103 (PMC11997465; doi:10.1002/ece3.71103)
Supplement: Supplementary file 1 — Appendix S1. [file ECE3-15-e71103-s001.docx]

**Supporting Information**

Additional Supporting Information may be found in the online version of this article:

**Table S1 Different primers for several plant barcoding regions**

| Gene | Primer | Direction | Sequence5’-3’ | Reference |  |
| --- | --- | --- | --- | --- | --- |
| rbcL | 1F | f | ATGTCACCACAAACAGAAAC | Li *et al*., 2011 | |
|  | 724R | r | TCGCATGTACCTGCAGTAGC |  |  |
|  | rbcLa-f | f | ATGTCACCACAAACAGAGACTAAAGC |  |  |
|  | rbcLa-rev | r | GTAAAATCAAGTCCACCRCG |  |  |
| matK | 3F_KIM | r | CGTACAGTACTTTTGTGTTTACGAG |  |  |
|  | 1R_KIM | f | ACCCAGTCCATCTGGAAATCTTGGTTC |  |  |
|  | Xf | f | TAATTTACGATCAATTCATTC |  |  |
|  | 5r | r | GTTCTAGCACAAGAAAGTCG |  |  |
|  | 472F | f | CCCRTYCATCTGGAAATCTTGGTTC | Jing *et al*., 2011 | |
|  | 1248R | r | GCTRTRATAATGAGAAAGATTTCTGC |  |  |
| ITS | ITS5A | f | CCTTATCATTTAGAGGAAGGAG | Cheng *et al*., 2016 | |
|  | ITS5 | f | GGAAGGAGAAGTCGTAACAAGG | Li *et al*., 2011 | |
|  | ITS4 | r | TCCTCCGCTTATTGATATGC |  |  |

**Table S2 Fossil-based minimum/maximum crown or stem group ages used in the dating of the phylogenetic tree.**

| Clade | Min/Max | Age (Mya) | | Reference |
| --- | --- | --- | --- | --- |
| CG Angiosperms | Min | 136.00 | Hughes & McDougall *et al*., 1987; Hughes *et al*., 1991; Magallón *et al*., 2013 | |
| CG Angiosperms | Max | 140.00 | Magallón *et al*., 2015 | |
| CG Eudicots | Min | 125.00 | Doyle *et al*., 1977; Leng & Friis, 2003, 2006; Magallón *et al*., 2013 | |
| SG Eudicots | Max | 135.60 | Doyle *et al*., 1977; Magallón *et al*., 2013 | |
| CG Rosaceae | Min | 89.80 | Mai,1995 | |
| SG Rhamnaceae | Min | 70.60 | Calvillo-Canadell & Cevallos-Ferriz, 2007; Magallón *et al*., 2015 | |
| SG Betulaceae | Min | 83.50 | Sims et al., 1999; Magallón *et al*., 2015 | |
| CG Fagales | Min | 96.00 | Pacltová, 1966; Magallón *et al*., 2013 | |
| CG Fabales | Min | 59.90 | Herendeen & Dilcher, 1992; Bell *et al*., 2010 | |
| SG Caprifoliaceae | Min | 36.00 | Manchester & Donoghue, 1995; Bell *et al.*, 2010 | |
| CG Oleaceae | Min | 5.33 | Barrón, 1992; Magallón *et al*., 2015 | |

Mya: million years ago; CG: crown group; SG: stem group

**Reference**

Magallón, S., Gómez‐Acevedo, S., Sánchez‐Reyes, L. L., & Hernández‐Hernández, T. (2015). A metacalibrated time‐tree documents the early rise of flowering plant phylogenetic diversity. *New Phytologist*, 207(2), 437-453.

Hughes, N. F. & McDougall, A. B. (1987). Records of angiospermid pollen entry into the English Early Cretaceous succession. *Rev. Palaeobot. Palyno,* 50, 255–272.

Hughes, N. F., McDougall, A. B., & Chapman, J. L. (1991). Exceptional new record of Cretaceous Hauterivian angiospermid pollen from southern England. *Journal of Micropalaeontology,* 10(1), 75-82.

Magallón, S., Hilu, K. W., & Quandt, D. (2013). Land plant evolutionary timeline: Gene effects are secondary to fossil constraints in relaxed clock estimation of age and substitution rates. *American Journal of Botany*, 100(3), 556-573.

Leng, Q. & Friis, E. M. (2003). Sinocarpus decussatus gen. et sp. nov., a new angiosperm with basally syncarpous fruits from the Yixian Formation of Northeast China. *Plant Systematics and Evolution*, 241, 77–88.

Doyle, J. A., Biens, P., Doerenkamp, A. & Jardiné, S. (1977). Angiosperm pollen from the pre-Albian Lower Cretaceous of equatorial Africa. *Bull. Cent. Rech. Explor.-Prod. Elf-Aquitaine*, *1*, 451-473.

Pacltová, B. (1966). Pollen grains of angiosperms in the Cenomanian Peruc Formation in Bohemia. *Palaeobotanist,* 15, 52–54.

Mai, D. H. (1995). Tertiäre Vegetationsgeschichte Europas: Gustav Fischer. *Jena, Stuttgart, New York*, 691.

Bell, C. D., Soltis, D. E., & Soltis, P. S. (2010). The age and diversification of the angiosperms re‐revisited. *American journal of botany*, *97*(8), 1296-1303.

Herendeen, P. S. & Dilcher, D. L. (1992). Advances in Legume Systematics, Part 4. *The Fossil Record*. (Kew: Royal Botanic Gardens, London).

Manchester, S. R., & Donoghue, M. J. (1995). Winged fruits of Linnaeeae (Caprifoliaceae) in the Tertiary of western North America: Diplodipelta gen. nov. *International Journal of Plant Sciences*, *156*(5), 709-722.

Barrón, E. (1992). Presencia de Fraxinus excelsior Linne (Oleaceae, Gentianales) en el Mioceno superior de la depresión Ceretana: implicaciones tafonóicas y paleoecológicas. *Rev. Esp. Paleontol*, 7, 101–108.

Calvillo-Canadell, L. & Cevallos-Ferriz, S. R. S. (2007). Reproductive structures of Rhamnaceae from the Cerro del Pueblo (Late Cretaceous, Coahuila) and Coatzingo (Oligocene, Puebla) Formations. *American Journal of Botany*, 94, 1658–1669.

Sims, H. J., Herendeen, P. S., Lupia, R., Christopher, R. A. & Crane, P. R. (1999). Fossil flower with Normapolles pollen from the Upper Cretaceous of southeastern North America. *Review of Palaeobotany and Palynology*, 06, 131–151.

**Table S3 PCR amplification and sequence (SEQ) results of 147 woody species, genera and families in Loess Plateau.**

|  | rbcL | | matK | | ITS | | rbcL+matK+ITS | |
| --- | --- | --- | --- | --- | --- | --- | --- | --- |
|  | PCR | SEQ | PCR | SEQ | PCR | SEQ | PCR | SEQ |
| Species (147), % | 100 | 100 | 97.76 | 100 | 100 | 94.56 | 97.76 | 93.2 |
| Genus (85), % | 100 | 100 | 96.47 | 100 | 100 | 91.76 | 96.47 | 89.41 |
| Family (41), % | 100 | 100 | 95.12 | 100 | 100 | 92.68 | 95.12 | 87.8 |

**Table S4 The sequences of taxa without vouchers were downloaded from GenBank.**

| Group | Family | Species | Accession Number |
| --- | --- | --- | --- |
| Angiosperms | Rosaceae | *Cotoneaster submultiflorus Popov* | FJ796944.1 |
| Angiosperms | Caprifoliaceae | *Lonicera tangutica Maxim* | FJ217851.1 |
| Angiosperms | Liliaceae | *Smilax stans Maxim.* | JF461368.1 |
| Angiosperms | Tiliaceae | *Tilia paucicostata Maxim.* | AF460198.1 |
| Angiosperms | Aceraceae | *Acer mono Maxim.* | U57775.1 |
| Angiosperms | Rosaceae | *Cotoneaster acutifolius Turcz.* | JQ392375.1 |
| Angiosperms | Fagaceae | *Quercus aliena Bl.* | KX838217.1 |
| Gymnosperms | Pinaceae | *Pinus tabuliformis Carr.* | JX173185.1 |
| Gymnosperms | Pinaceae | *Pinus tabuliformis Carr.* | GQ865728.1 |
| Gymnosperms | Cupressaceae | *Platycladus orientalis (L.) Franco* | HM024067.1 |
| Gymnosperms | Cupressaceae | *Juniperus rigida Siebold & Zucc.* | AB030136.1 |
| Gymnosperms | Pinaceae | *Pinus tabuliformis Carr.* | AB161015 |

**Table S5 Phylogenetic structures (Mean ± SD) of whole community based on phylogeny resolved at the species level (barcode) and phylogeny resolved at the genus level with species being attached to their respective genera as basal polytomies. All the reginal species were** **regarded as the species pool (n=147). Bold indicates that the NRI/NTI is significantly different from null models (P<0.05).**

| Species level | The net relatedness index (NRI) | | | | | The net nearest taxa index (NTI) | | | | | |  |
| --- | --- | --- | --- | --- | --- | --- | --- | --- | --- | --- | --- | --- |
|  | 100 | 400 | | 2500 | | 100 | | 400 | | 2500 | |  |
| Abundance weighted | 0.07±0.61 | 0.03±0.59 | | -0.09±0.59 | | 0.07±0.56 | | 0.01±0.59 | | -0.14±0.68 | |  |
| Occurrence based | 0.01±0.97 | -0.01±1.03 | | 0.10±1.14 | | -0.01±0.96 | | -0.02±1.08 | | 0.04±1.21 | |  |
| Genus level | The net relatedness index (NRI) | | | | | | The net nearest taxa index (NTI) | | | | | |
|  | 100 | | 400 | | 2500 | | 100 | | 400 | | 2500 | |
| Abundance weighted | **0.58±0.77** | | **0.51±0.62** | | **0.84±0.69** | | **0.85±0.85** | | **0.81±1.01** | | **0.75±0.53** | |
| Occurrence based | **0.57±0.76** | | **0.49±0.61** | | **0.68±0.64** | | **0.86±0.85** | | **0.79±1.00** | | **0.67±0.68** | |

**Table S6 Phylogenetic structures (Mean ± SD) of whole community based on phylogeny resolved at the species level (barcode) and phylogeny resolved at the genus level with species being attached to their respective genera as basal polytomies. All the plot species were regarded as the species pool (n=51). Bold indicates that the NRI/NTI is significantly different from null models (P<0.05).**

| Species level | The net relatedness index (NRI) | | | | | The net nearest taxa index (NTI) | | | | | |  |
| --- | --- | --- | --- | --- | --- | --- | --- | --- | --- | --- | --- | --- |
|  | 100 | 400 | | 2500 | | 100 | | 400 | | 2500 | |  |
| Abundance weighted | -0.04+0.55 | -0.09+0.5 | | **-0.78+0.42** | | 0.02+0.52 | | -0.16+0.59 | | **-0.65+0.70** | |  |
| Occurrence based | 0.01+0.52 | -0.16+0.59 | | -0.65+0.71 | | -0.09+0.81 | | -0.11+1.05 | | -0.17+0.86 | |  |
| Genus level | The net relatedness index (NRI) | | | | | | The net nearest taxa index (NTI) | | | | | |
|  | 100 | | 400 | | 2500 | | 100 | | 400 | | 2500 | |
| Abundance weighted | **0.48±0.68** | | **0.41±0.69** | | **0.88±0.75** | | **0.71±0.87** | | **0.61±1.11** | | 0.47±0.74 | |
| Occurrence based | **0.49±0.79** | | **0.43±0.70** | | **0.72±0.81** | | **0.72±0.88** | | **0.61±1.19** | | 0.38±0.92 | |

**Table S7 The scale-dependency of functional structure (Mean ± SD) for whole community and different vertical structural layers based on the reginal species pool (n=147)**

| Traits |  | Functional structure  Abundance weighted | | | Functional structure  Occurrence based | | |
| --- | --- | --- | --- | --- | --- | --- | --- |
|  | Scales(m^2^) | 100 | 400 | 2500 | 100 | 400 | 2500 |
| SLA | Whole community | -0.84±0.84^a^ | -0.86±0.76^a^ | -1.00±0.86^a^ | -0.97±0.82^a^ | -0.89±0.69^a^ | -0.54±0.6^a^ |
|  | Trees | -0.83±0.70^a^ | -0.92±0.65^a^ | -0.93±0.77^a^ | -0.9±0.72^a^ | -0.93±0.62^a^ | -0.77±0.86^a^ |
|  | Shrubs | -0.33±0.83^a^ | -0.29±0.83^a^ | -0.20±1.00^a^ | **-0.5±0.79^b^** | **-0.4±0.8^b^** | **-0.03±0.64^a^** |
| Seed Mass | Whole community | 0.98±0.71^a^ | 1.03±0.73^a^ | 1.25±0.79^a^ | 0.72±0.79^a^ | 0.7±0.6^a^ | 0.61±0.29^a^ |
|  | Trees | 1.10±0.40^a^ | 1.14±0.36^a^ | 1.28±0.18^a^ | **1.06±0.64^b^** | **1.4±0.55^a^** | **1.57±0.19^a^** |
|  | Shrubs | **-0.44±0.07^a^** | **-0.53±0.08^b^** | **-0.60±0.08^b^** | **-0.5±0.08^a^** | **-0.7±0.07^b^** | **-0.88±0.04^b^** |
| Height | Whole community | 1.32±0.80^a^ | 1.31±0.72^a^ | 1.64±0.68^a^ | **0.48±0.91^a^** | **0.12±0.58^b^** | **0.19±0.41^b^** |
|  | Trees | 0.78±0.52^a^ | 0.81±0.35^a^ | 0.91±0.13^a^ | 0.58±0.61^a^ | 0.62±0.47^a^ | 0.33±0.17^a^ |
|  | Shrubs | **-1.76±0.41^a^** | **-2.21±0.39^b^** | **-2.54±0.39^b^** | **-2.06±0.45^a^** | **-3.1±0.4^b^** | **-3.92±0.13^c^** |
| All traits | Whole community | 0.82±0.56^a^ | 0.84±0.54^a^ | 1.02±0.50^a^ | 0.63±0.77^a^ | 0.62±0.61^a^ | 0.52±0.29^a^ |
|  | Trees | 1.30±0.47^a^ | 1.37±0.42^a^ | 1.54±0.27^a^ | **0.99±0.61^a^** | **1.31±0.48^b^** | **1.51±0.22^b^** |
|  | Shrubs | **-0.45±0.07^a^** | **-0.53±0.08^b^** | **-0.60±0.08^b^** | **-0.49±0.07^a^** | **-0.67±0.06^b^** | **-0.83±0.04^c^** |

Annotation: SLA, specific leaf area; All traits, the three traits selected in this study. Different lowercase letters in black bold represent significant differences in the phylogenetic structures among different spatial scales.

**Table S8 The scale-dependency of functional structure (Mean ± SD) for whole community and different vertical structural layers based on the reginal species pool (n=51)**

| Traits |  | Functional structure  Abundance weighted | | | Functional structure  Occurrence based | | |
| --- | --- | --- | --- | --- | --- | --- | --- |
|  | Scales(m^2^) | 100 | 400 | 2500 | 100 | 400 | 2500 |
| SLA | Whole community | -0.74±0.66^a^ | -0.82±0.61^a^ | -0.83±0.73^a^ | -0.81±0.7^a^ | -0.84±0.62^a^ | -0.71±0.86^a^ |
|  | Trees | -0.74±0.65^a^ | -0.82±0.6^a^ | -0.82±0.71^a^ | -0.82±0.68^a^ | -0.86±0.61^a^ | -0.74±0.87^a^ |
|  | Shrubs | -0.30±0.79^a^ | -0.26±0.79^a^ | -0.42±0.95^a^ | -0.42±0.76^a^ | -0.3±0.78^a^ | 0.06±0.65^a^ |
| Seed Mass | Whole community | 1.51±0.54^a^ | 1.60±0.43^a^ | 1.77±0.18^a^ | **1.55±1.05^b^** | **2.10±0.90^a^** | **2.40±0.42^a^** |
|  | Trees | 1.56±0.59^a^ | 1.63±0.48^a^ | 1.85±0.23^a^ | **1.54±1.02^b^** | **2.08±0.90^a^** | **2.40±0.42^a^** |
|  | Shrubs | -0.73±0.13^a^ | -0.91±0.13^a^ | -1.04±0.15^a^ | **-0.85±0.15^a^** | **-1.25±0.14^b^** | **-1.70±0.03^c^** |
| Height | Whole community | 1.28±0.54^a^ | 1.38±0.36^a^ | 1.51±0.28^a^ | 1.14±0.55^a^ | 1.4±0.40^a^ | 1.33±0.15^a^ |
|  | Trees | 1.18±0.48^a^ | 1.26±0.31^a^ | 1.39±0.24^a^ | 1.2±0.59^a^ | 1.43±0.41^a^ | 1.33±0.17^a^ |
|  | Shrubs | -1.19±0.30^a^ | -1.48±0.28^a^ | -1.70±0.27^a^ | **-1.42±0.33^a^** | **-2.19±0.32^b^** | **-2.92±0.09^b^** |
| All traits | Whole community | 1.47±0.54^a^ | 1.56±0.43^a^ | 1.74±0.19^a^ | **1.45±1.01^b^** | **2.03±0.88^a^** | **2.36±0.41^a^** |
|  | Trees | 1.42±0.54^a^ | 1.51±0.44^a^ | 1.69±0.22^a^ | **1.49±1.01^b^** | **2.06±0.88^a^** | **2.41±0.40^a^** |
|  | Shrubs | -0.77±0.13^a^ | -0.95±0.13^a^ | -1.10±0.14^a^ | **-0.86±0.15^a^** | **-1.26±0.14^b^** | **-1.69±0.10^c^** |

Annotation: SLA, specific leaf area; All traits, the three traits selected in this study. Different lowercase letters in black bold represent significant differences in the phylogenetic structures among different spatial scales.


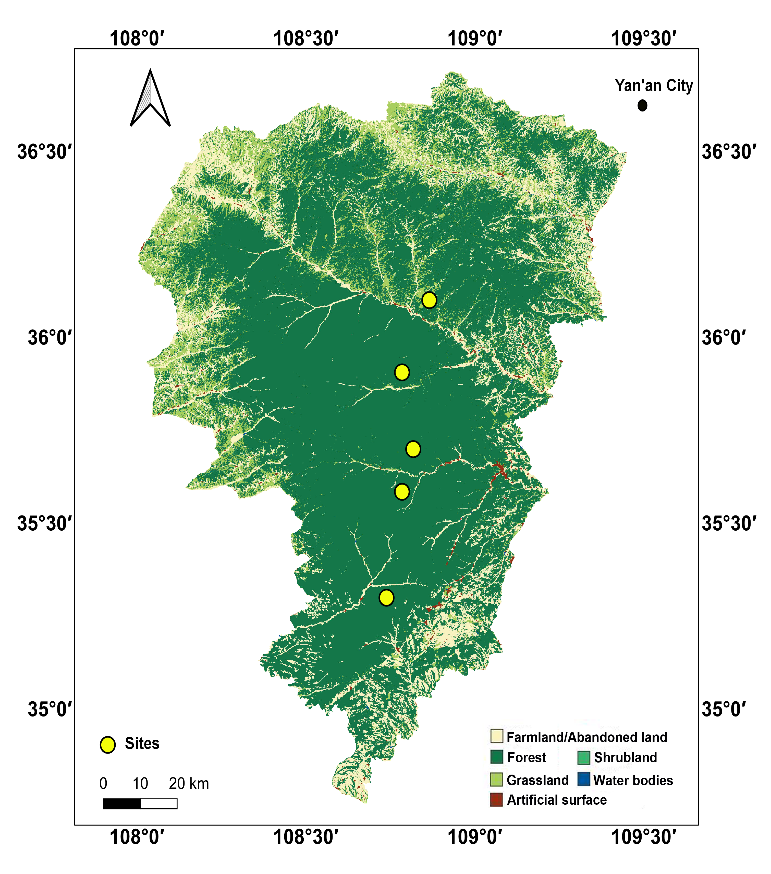


**Fig. S1.** Forest boundary and land-cover map of Ziwuling in Loess Plateau. The site distribution covers the main area of *Quercus* forest. Land-cover map is based on MODIS-NDVI, normalized difference vegetation index. The dataset of forest boundary is provided by National Cryosphere Desert Data Center. (http://www.ncdc.ac.cn).


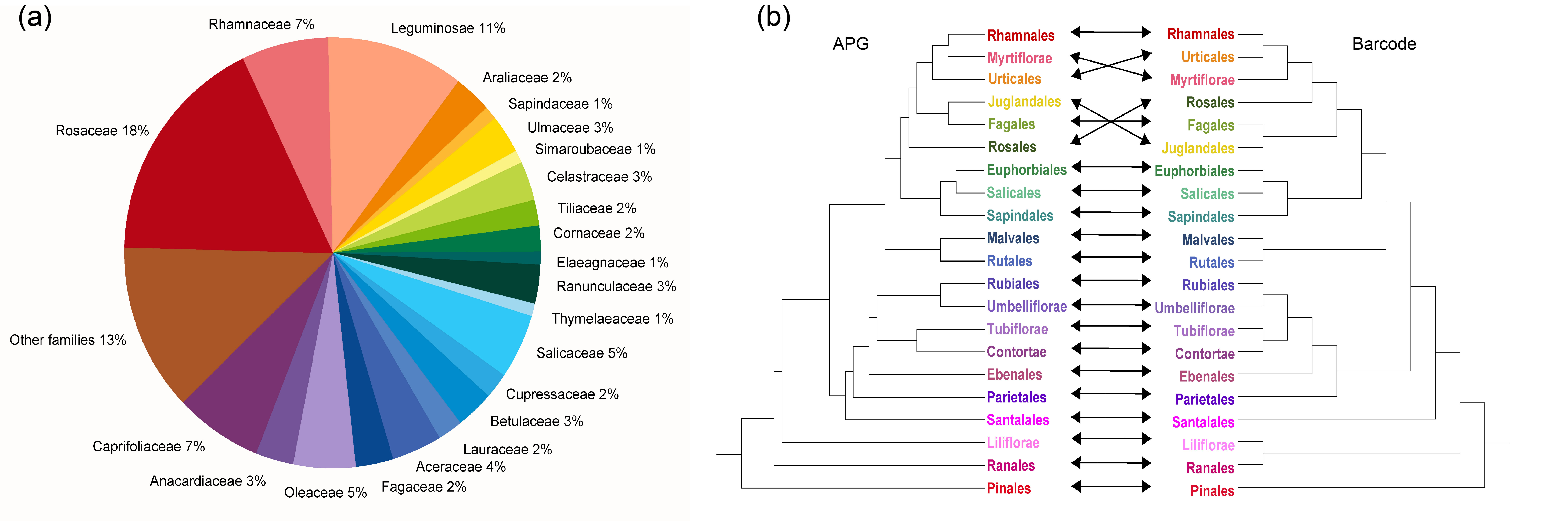


**Fig. S2.** Species abundance of 41 plant families for 147 species in the Loess Plateau (a). Comparison of ordinal-level topologies of the trimmed Phylomatic tree obtained by APG III (R20120829) (APG) and the barcoding tree (rbcL + matK + ITS; Barcode) using maximum likelihood analysis (b).


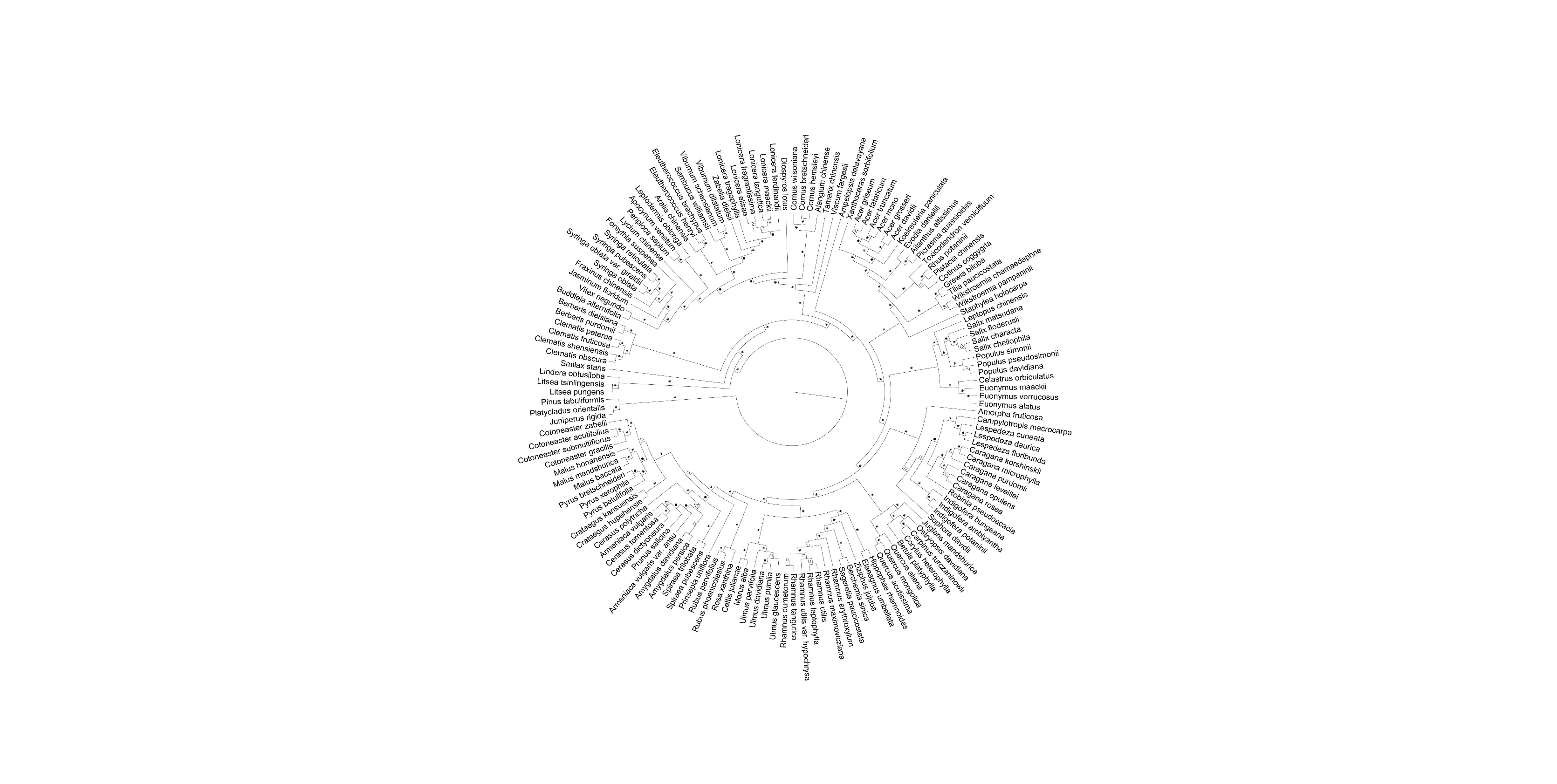


**Fig. S3.** The maximum likelihood tree of 147 woody plants in Loess Plateau based on combination of rbcL, matK and ITS sequences using the APG IV as the guide or constraint tree. Nodes with strong ratchet support (≥85%) are indicated by an asterisk and nodes with moderate (>70%-85%) or weak (>50%-70%) support is indicated once by an open triangle or open square, while solid black dot for nodes with poor (<50%) support.
